# Supplementary material for: The genomic origins and evolutionary path to a key innovation in the world’s most venomous snakes
Source: bioRxiv. 2026 Jun 28:2026.06.23.733212. Preprint. [Version 1] doi: 10.64898/2026.06.23.733212 (PMC13320979; doi:10.64898/2026.06.23.733212)
Supplement: Supplement 1 [file media-1.pdf]

The genomic origins and evolutionary path to a key innovation in the world's most venomous snakes

Jory van Thiel, Noah L. Dowell, Cara F. Smith, Elda E. Sanchez, and Sean B. Carroll

Correspondence and request for materials should be addressed to Sean B. Carroll
Email: carrolls@hhmi.org.

This PDF file includes:

Supporting text
Figures S1 to S16
Tables S1 to S2
SI References

Supporting text

Short-read RNA-seq mapping

To search for evidence of *F10* and *F5* expression in *P. textilis* venom gland tissue, we mapped short RNA-seq reads to the corresponding genomic loci. Adult *P. textilis* venom gland RNA-seq data were

retrieved from the SRA database (accession SRR33084395). Low-quality reads and adapter sequences were removed using Trimmomatic (version 0.40-rc1) (1) with the following parameters: “ILLUMINACLIP:TruSeq3-PE-2.fa:2:30:10”, “LEADING:5”, “TRAILING:5”, “SLIDINGWINDOW:4:30”, and “MINLEN:40”. Read quality was subsequently assessed, and adapter removal was confirmed using FastQC (version 0.12.1). The processed RNA-seq reads were aligned to all contigs in our *P. textilis* genome assembly using the BWA-MEM algorithm (2). Using the Samtools “view” command (3), paired-end reads with a mapping quality (MAPQ) score greater than 20 were retained and sorted, and only the reads mapping to the two contigs containing the *F10* and *F5* genes were extracted. Mapped reads were subsequently visualized and inspected using Integrative Genomics Viewer (IGV; version 2.16.0) (4, 5) to search for reads mapping to regions unique to the *F10* or *F5* genes and not to their respective paralogs.

Figures

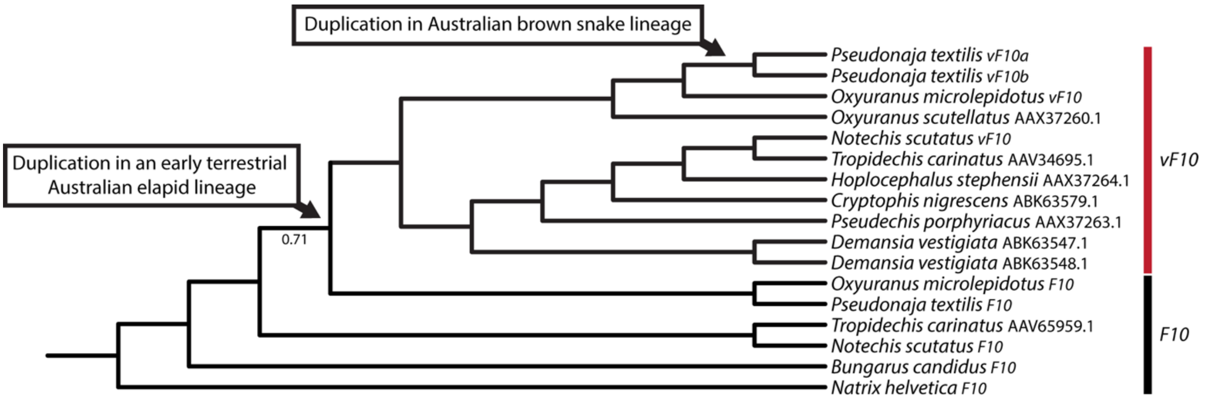

Fig. S1. A venom-expressed *F10* gene was duplicated in the brown snake lineage (*Pseudonaja* spp.). A protein phylogeny based on hypothetical translations of the *F10* genes and full-length cDNA sequences from distinct Australian elapid species. At the base of the phylogeny, there is a cluster of *F10* genes (encoding the hemostatic protein), and nested within it, a well-supported monophyletic clade consisting of *vF10* (encoding the venom toxin). In the venom gene clade, the *vF10b* gene clusters with *vF10a*, indicating that *vF10b* arose through duplication of the venom-expressed *vF10a* and not from the liver-expressed *F10* gene, a scenario that has previously been proposed (6). The phylogeny was rooted with *N. helvetica F10* protein sequence as the outgroup. Only Bayesian posterior probabilities below 1.00 are shown.

	1 76
Pseudonaja textilis vF10a	MAPQLLLCLILTFLWSLPEAESNVFLKSKVANRFLQRTKRANSLVEEFKSGNIERECIEERCSKEEAREVFEDDEK
Pseudonaja textilis vF10b	MAPQLLLCLILTFLWSLPEAESNVFLKSKVANRFLQRTKRANSLVEEFKSGNIERECIEERCSKEEAREVFEDDEK
Oxyuranus microlepidotus vF10	MAPQLLLCLILTFLWSLPEAESNVFLKSKVANRFLQRTKRANSLFEEFRSGNIERECIEERCSKEEAREVFEDDEK
Notechis scutatus vF10	MAPQLLLCLILTFLWSLPEAESNVFLKSKVANRFLQRTKRSNSLFEEIRPGNIERECIEEKCSKEEAREVFEDNEK
Pseudonaja textilis F10	MAPQLLLCLILTFLWSLPEAESNVFLKSKVANRFLQRTKRANSLFEEFKPGNIERECIEERCSKEEAREAFEDDEK
Oxyuranus microlepidotus F10	MAPQLLLCLILTFLWSLPEAESNVFLKSKVANRFLQRTKRANSLFEEFKPGNIERECIEERCSKEEAREAFEDDEK
Notechis scutatus F10	MAPQLLLCLILTFLWSLPEAESNVFLKSKVANRFLQRTKRANSLFEEFKAGNIERECIEERCSKEEAREAFEDNEK
Aipysurus laevis F10	MAPQLLLCLILTFLWSLPEAESNVFLKSKVANRFLQRTKRANSLFEEFKAGNIERECIEERCSKEEAREAFEDNEK
Hydrophis major F10	MAPQLLLCLIFTFLWSLPEAESNVFLKSKVANRFLQRTKRANSLFEEFKAGNIERECIEERCSKEEAREAFEDNEK
Hydrophis elegans F10	MAPQLLLCLIFTFLWSLPEAESNVFLKSKVANRFLQRTKRANSLFEEFKAGNIERECIEERCSKEEAREAFEDNEK
Hydrophis curtus F10	MAPQLLLCLIFTFLWSLPEAESNVFLKSKVANRFLQRTKRANSLFEEFKAGNIERECIEERCSKEEAREAFEDNEK
Hydrophis ornatus F10	MAPQLLLCLIFTFLWSLPEAESNVFLKSKVANRFLQRTKRANSLFEEFKAGNIERECIEERCSKEEAREAFEDNEK
Bungarus candidus F10	MAPQLLLCLILTFLWSLPEAESNVFLKSKVANRFLQRTKRANSLFEEIKPGNIERECREERCSKEEAREAFENNEE
Natrix helvetica F10	MAPQLLLCLILTFLWSLPNAESNVFLKGKVANRFLQRTKRANSIFEEFKAGNIERECIEERCSKEEAREAFEDQEK

	77 152
Pseudonaja textilis vF10a	TETFWNVYVDGDQCSSNPCHYRGICKDGIGSYTCTCLSGYEGKNCERVLYKSCRVDNGNCWHFCKSVQNDIQCSCA
Pseudonaja textilis vF10b	TETFWNVYVDGDQCSSNPCHYRGICKDGIGSYTCTCLSGYEGKNCERVLYKSCRVDNGNCWHFCKHVQNDIQCSCA
Oxyuranus microlepidotus vF10	TETFWNVYVDGDQCSSNPCHYRGTCKDGIGSYTCTCLFGYEGKNCERVLYKSCRVDNGNCWHFCKPVQNDIQCSCA
Notechis scutatus vF10	TETFWNVYVDGDQCSSNPCHYRGTCKDGIGSYTCTCLPNYEGKNCEKVLYQSCRVDNGNCWHFCKRVQSETQCSCA
Pseudonaja textilis F10	TETFWNVYVDGDQCSSNPCHYGGTCKDGIGSYTCTCLSGYEGKNCEYVLYKSCRVDNGDCWHFCKPVQNGIQCSCA
Oxyuranus microlepidotus F10	TETFWNVYVDGDQCSSNPCHYGGTCKDGIGSYTCTCLSGYEGKNCEYVLYKSCRVDNGNCWHFCKPVQNGIQCSCA
Notechis scutatus F10	TETFWNVYVDGDQCSSNPCHYGGTCKDGIGSYTCTCLAGYEGKNCQYVLYQSCRVDNGNCWHFCKRVQSEIQCSCA
Aipysurus laevis F10	TETFWNVYVDGDQCSSNPCHYGGTCKDGIGSYTCTCLAGYEGKNCQYVLYKSCRVDNGNCWHFCKPVQNEIQCSCA
Hydrophis major F10	TETFWNVYVDGDQCSSNPCHYGGTCKDGIGSYTCTCLAGYEGKNCQYVLYKSCRVDNGNCWHFCKPVQNEIQCSCA
Hydrophis elegans F10	TETFWNVYVDGDQCSSNPCHYGGTCKDGIGSYTCTCLAGYEGKNCQYVLYKSCRVDNGNCWHFCKPVQNEIQCSCA
Hydrophis curtus F10	TETFWNVYVDGDQCSSNPCHYGGTCKDGIGSYTCTCLAGYEGKNCQYVLYKSCRVDNGNCWHFCKPVQNEIQCSCA
Hydrophis ornatus F10	TETFWNVYVDGDQCSSNPCHYGGTCKDGIGSYTCTCLAGYEGKNCQYVLYKSCRVDNGNCWHFCKPVQNEIQCSCA
Bungarus candidus F10	TEKFWNIYVDGDQCSSNPCHYGGTCKDGIGSYTCTCLAGYQGKNCEYIIYKSCRVDNGNCWHFCKLVQSEIQCSCA
Natrix helvetica F10	TENFWNVYVDGDQCLSNPCHYGATCKDGIGNFSCTCLAGYQGKTCESFILRSCRLDNGNCWHFCKSDQNKIHCSCA

	153 228
Pseudonaja textilis vF10a	EGYLLGEDGHSCVAGGNFSCGRNIKTRNKR**EASLPDF-----------------------------VQSHNATLLK**
Pseudonaja textilis vF10b	EGYLLGEDGHSCVAGGNFSCGRNIKTRNKR**EANLPDF-----------------------------VQSQNATLLK**
Oxyuranus microlepidotus vF10	EGYLLGEDGHSCVAGGNFSCGRNIKTRNKR**EASLPDF-----------------------------VQSQNATLLK**
Notechis scutatus vF10	ESYRLGVDGHSCVAEGDFSCGRNIKARNKR**EASLPDF-----------------------------VQSQKATLLK**
Pseudonaja textilis F10	ESYLLGEDGHSCVAGGDFSCGRNIKTRNKR**EANLPDFQTDFSDDYDEIDENNFVETPTNFSGLVLTVQSQNATLLK**
Oxyuranus microlepidotus F10	ESYLLGEDGHSCVAGGDFSCGRNIKTRNKR**EANLPDFQTDFSDDYDEIDENNFVETPTNFSGLVPTVQSQNATLLK**
Notechis scutatus F10	ESYLLGDDGHSCVAGGDFSCGRNIKARNKR**EASLPDFQTDFSDDYDAIDENNFVETPTNFSGLVPTVQSQNATLLK**
Aipysurus laevis F10	ESYLLGDDGYSCVAEDDFSCGRNIKARNKR**EASLPDFQTDFSDDYDAIDENNFVETPTNFSGLVPTVQSQNASLLK**
Hydrophis major F10	ESYLLGDDGYSCVAEDDFSCGKNIKARNKR**EASLPDFQTDFSDDYDAIDENNFVETPTNFSGLVPTVQSQNANLLK**
Hydrophis elegans F10	ESYLLGDDGYSCVAEDDFSCGKNIKARNKR**EASLPDFQTDFSDDYDAIDENNFVETPTNFSGLVPTVQSQNANLLK**
Hydrophis curtus F10	ESYLLGDDGYSCVAEDDFSCGKNIKARNKR**EASLPDFQTDFSDDYDAIDENNFVETPTNFSGLVPTVQSQNANLLK**
Hydrophis ornatus F10	ESYLLGDDGYSCVAEDDFSCGKNIKARNKR**EASLPDFQTDFSDDYDAIDENNFVETPTNFSGLVPTVQSQNANLLK**
Bungarus candidus F10	ESYVLGDDEYSCVAEDDFSCGRNIKARNKR**EASLPDFQTEFSEDYDAIDENNFVETSTNFSSLVPTLQSRNATSLK**
Natrix helvetica F10	EGYILGDDGQSCVAGGDFSCGRNIKARNKR**EASLVDFQEDFSDDYDVIEEESFVETPTNFSSLVPIMQHRNATKPR**

	229 304
Pseudonaja textilis vF10a	**KSDNPSPDIR**IVNGMDCKLGECPWQAALVDDKKGVFCGGTILSPIYVLTAAHCINETETISVVV-GEIDRSRAETG
Pseudonaja textilis vF10b	**KSDNPSPDIR**IVNGMDCKLGECPWQAALVDEKEGVFCGGTILSPIYVLTAAHCINETETISVVV-GEIDKSRIETG
Oxyuranus microlepidotus vF10	**KSDNPSPDIR**IVNGMDCKLGECPWQAVLVDEKEGVFCGGTILSPIYVLTAAHCINQTEKISVVV-GEIDKSRVETG
Notechis scutatus vF10	**KSDNPSPDIR**IVNGMDCKLGECPWQAVLINEKGEVFCGGTILSPIHVLTAAHCINQTKSVSVIV-GEIDISRKETR
Pseudonaja textilis F10	**KSDNPSPDIR**VVNGTDCKLGECPWQALLLNDEGDGFCGGTILSPIYVLTAAHCINQTKYITVVV-GEIDISSKKTG
Oxyuranus microlepidotus F10	**KSDNPSPDIR**VVNGTDCKLGECPWQALLINDQGDGFCGGTILSPIYVLTAAHCINQTKYIRVVV-GEIDISRKKTG
Notechis scutatus F10	**KSDNPSPDIR**VVNGTDCKLGECPWQALLINDQGDGFCGGTILSPIYVLTAAHCINQTKYIRVVV-EEIDISRKETR
Aipysurus laevis F10	**KSDNPSPDIR**VVNGTDCKLGECPWQALLINDQGDGFCGGTILSPIYVLTAAHCINQTKYIRVVV-GEIDISRKKTG
Hydrophis major F10	**KSDNPSPDVR**VVNGTDCKQGECPWQVLLINDRGDGFCGGTILSPIYVLTAAHCINQTKYIRVVVAGEIDISRKKTG
Hydrophis elegans F10	**KSDNPSPDIR**VVNGTDCKQGECPWQALLINDRGDGFCGGTILSPIYVLTAAHCINQTKYIRVVV-GEIDISRKKTG
Hydrophis curtus F10	**KSDNPSPDIR**VVNGTDCKQGECPWQALLINDRGDGFCGGTILSPIYVLTAAHCINQTKYIRVVV-GEIDISRKKTG
Hydrophis ornatus F10	**KSDNPSPDIR**VVNGTDCKQGECPWQALLINDRGDGFCGGTILSPIYVLTAAHCINQTKYIRVVV-GEIDISRKKTG
Bungarus candidus F10	**KSNNPSPEIR**VVNGTDCKLGECPWQALLLNDQGDGFCGGTILSSIYVLTAAHCINQTKHITVVV-GEVDISSKRNG
Natrix helvetica F10	**KSDNSNPEGR**VVNGTDCKPGECPWQALLINDQGDGFCGGTVLSPMYVLTAAHCINQTTRIKVVV-GEVDISSKKTG

```
                                305                                                                       380
Pseudonaja textilis vF10a       PLLSVDKVYVHKKFVPPKKSQEFYEKFDLVSYDYDIAIIQMKTPIQFSENVVPACLPTADFANQVLMKQDFGIVSG
Pseudonaja textilis vF10b       PLLSVDKIYVHKKFVPPQKAY----KFDLAAYDYDIAIIQMKTPIQFSENVVPACLPTADFANQVLMKQDFGIVSG
Oxyuranus microlepidotus vF10   HLLSVDKIYVHKKFVPPKKGYKFYEKFDLVSYDYDIAIIQMKTPIQFSENVVPACLPTADFANQVLMKQDFGIISG
Notechis scutatus vF10          RLLSVDKIYVHTKFVPPNYYY-VHQNFDRVAYDYDIAIIRMKTPIQFSENVVPACLPTADFANEVLMKQDSGIVSG
Pseudonaja textilis F10         RLHSVDKIYVHQKFVP-------------ATYDYDIAIIQLKTPIQFSENVVPACLPTADFANQVLMKQNFGIVSG
Oxyuranus microlepidotus F10    RLLSVDKIYVHQKFVP-------------ATYDYDIAIIQLKTPIQFSENVVPACLPTADFANQVLMKQNFGIVSG
Notechis scutatus F10           RLLSVDKIYVHQKFVP-------------STYDYDIALIQMKTPIQFSENVVPACLPTADFANQVLMKQDFGIVSG
Aipysurus laevis F10            PL-SVDKIYVHKNFVP-------------TTYDYDIALIQLKTAIQFSENVVPACLPTADFANQVLMKQDFGIVSG
Hydrophis major F10             -LLSVDKIYVHKNFVP-------------TTYDYDIALIQLKTAIQFSENVVPACLPTADFANQVLMKQDFGIVSG
Hydrophis elegans F10           -LLSVDKIYVHKNFVP-------------TTYDYDIALIQLKTAIQFSENVVPACLPTADFANQVLMKQDFGIVSG
Hydrophis curtus F10            -LLSVDKIYVHKNFVP-------------TTYDYDIALIQLKTAIQFSENVVPACLPTADFANQVLMKQDFGIVSG
Hydrophis ornatus F10           -LLSVDKIYVHKNFVP-------------TTYDYDIALIQLKTAIQFSENVVPACLPTADFANQVLMKQDFGIVSG
Bungarus candidus F10           RLIAVDKIYVHQKFVP-------------ATYDYDIAIIQLKTPIQFSENVVPACLPTADFANQVLMKQNFGIVSG
Natrix helvetica F10            HLLAVNKIYVHQKFVL-------------ATYDYDIAIIQLKTPIRFSENVIPACLPTADFANQVLMKQDSGVISG

                                381                                                                       456
Pseudonaja textilis vF10a       FGGIFERGPNSKTLKVLKVPYVDRHTCMLSSNFPITPTMFCAGYDTLPQDACQGDSGGPHITAYRDTHFITGIVSW
Pseudonaja textilis vF10b       FGRIFEKGPKSKTLKVLKVPYVDRHTCMVSSETPITPNMFCAGYDTLPRDACQGDSGGPHTTVYRDTHFITGIVSS
Oxyuranus microlepidotus vF10   FGRIFEKGPKSNTLKVLKVPYVDRHTCMVSSESPITPTMFCAGYDTLPRDACQGDSGGPHITAYRDTHFITGIVSW
Notechis scutatus vF10          FGRIRFKEPTSNTLKVITVPYVDRHTCMLSSDFRITQNMFCAGYDTLPQDACQGDSGGPHITAYRDTHFITGIISW
Pseudonaja textilis F10         FGRTRERGKTSNTLKVVTLPYVDRHTCMLSSNFPITQNMFCAGYDTLPQDACQGDSGGPHITAYRDTHFITGIVSW
Oxyuranus microlepidotus F10    FGRTRERGQTSNTLKVVTLPYVDRHTCMLSSNFPITQNMFCAGYDTLPQDACQGDSGGPHITAYRDTHFITGIVSW
Notechis scutatus F10           FGRIRERGQTSNTLKVITLPYVDRHTCMLSSNFPITQNMFCAGYNTLPQDACQGDSGGPHITAYRDTHFITGIISW
Aipysurus laevis F10            FGRTRERGQTSKTLKVVTLPYVDRHTCMLSSNFPITQNMFCAGYNTLPQDACQGDSGGPHITAYRDTHFITGIISW
Hydrophis major F10             FGRTRERGKTSNTLKVVTLPYVDRHTCMLSSNFPITQNMFCAGYNTLPQDACQGDSGGPHITAYRDTHFITGIISW
Hydrophis elegans F10           FGRTRERGKTSNTLKVVTLPYVDRHTCMLSSNFPITQNMFCAGYNTLPQDACQGDSGGPHITAYRDTHFITGIISW
Hydrophis curtus F10            FGRTRERGKTSNTLKVVTLPYVDRHTCMLSSNFPITQNMFCAGYNTLPQDACQGDSGGPHITAYRDTHFITGIISW
Hydrophis ornatus F10           FGRTRERGKTSNTLKVVTLPYVDRHTCMLSSNFPITQNMFCAGYNTLPQDACQGDSGGPHITAYRDTHFITGIISW
Bungarus candidus F10           FGRTRERGRTSNTLKVVTLPYVDRHICKISSNFPITENMFCAGYNTLPQDACQGDSGGPHITAYRDTHFITGIISW
Natrix helvetica F10            FGRIKERGRTSNTLKMVTLPYVDRHVCKLSSNFPITENMFCAGYRTLAQDACQGDSGGPHVTAYSDTYFVTGIISW

                                457                                     497
Pseudonaja textilis vF10a       GEGCARKGRYGIYTKLSKFIPWIKRIMRQKLPSTESSTGRL
Pseudonaja textilis vF10b       GEGCARNGKYGIYTKLSKFIPWIKRIMRQKLPSTESSTGRL
Oxyuranus microlepidotus vF10   GEGCAKKGKYGIYTKVSKFILWIKRIMRQKLPSTESSTGRL
Notechis scutatus vF10          GEGCARKGKYGVYTKVSRFIPWIKKIMSLK-----------
Pseudonaja textilis F10         GEGCAQTGKYGVYTKVSKFILWIKRIIRQKQPSTESSTGQL
Oxyuranus microlepidotus F10    GEGCAQTGKYGVYTKVSKFILWIKRIMRQKLPSTESSTGRL
Notechis scutatus F10           GEGCAQTGKYGVYTKVSRFILWIKRIMRLKLPSTESSTGRL
Aipysurus laevis F10            GEGCAQTGKYGVYTKVSRFILWIKRIMRLKIPSTE------
Hydrophis major F10             GEGCAQTGKYGVYTKVSRFILWIKRIMRLKIPSTESSTGRL
Hydrophis elegans F10           GEGCAQTGKYGVYTKVSRFILWIKRIMRLKIPSTESSTGRL
Hydrophis curtus F10            GEGCAQTGKYGVYTKVSRFILWIKRIMRLKTPSTESSTGRL
Hydrophis ornatus F10           GEGCAQTGKYGVYTKVSRFILWIKRIMRLKIPSTESSTGRL
Bungarus candidus F10           GEGCAQPDKYGVYTKVSKFIPWIKRIMRQKLPGTDSSSGRL
Natrix helvetica F10            GEGCAQKDKYGVYTKVSKFIPWIKRIMRQKVPRTESGTARP
```

Fig. S2. The venom factor X proteins have a truncated activation peptide region. Amino acid sequence alignment of hypothetical protein translations of *F10* genes**.** Residues shown in grey encode the signal peptide and propeptide regions, and residues in bold indicate the activation peptide region, which is intact in proteins encoded by the *F10* genes but exhibits a 29-amino-acid deletion in proteins encoded by *vF10* genes.

Oxyuranus microlepidotus vF10	MAPQLLLCLILTFLWSLPEAESNVFLKSKVANRFLQRTKRANSLFEEF**RS**GNIERECIEERCSKEEARE**V**FEDDEK
Oxyuranus microlepidotus F10	MAPQLLLCLILTFLWSLPEAESNVFLKSKVANRFLQRTKRANSLFEEF**KP**GNIERECIEERCSKEEARE**A**FEDDEK
transcript/802606	MAPQLLLCLILTFLWSLPEAESNVFLKSKVANRFLQRTKRANSLFEEF**KP**GNIERECIEERCSKEEARE**A**FEDDEK
Oxyuranus microlepidotus vF10	TETFWNVYVDGDQCSSNPCHY**R**GTCKDGIGSYTCTCL**F**GYEGKNCE**R**VLYKSCRVDNGNCWHFCKPVQN**D**IQCSCA
Oxyuranus microlepidotus F10	TETFWNVYVDGDQCSSNPCHY**G**GTCKDGIGSYTCTCL**S**GYEGKNCE**Y**VLYKSCRVDNGNCWHFCKPVQN**G**IQCSCA
transcript/802606	TETFWNVYVDGDQCSSNPCHY**G**GTCKDGIGSYTCTCL**S**GYEGKNCE**Y**VLYKSCRVDNGNCWHFCKPVQN**G**IQCSCA
Oxyuranus microlepidotus vF10	E**G**YLLGEDGHSCVAGG**N**FSCGRNIKTRNKREA**S**LPDF-----------------------------VQSQNATLLK
Oxyuranus microlepidotus F10	E**S**YLLGEDGHSCVAGG**D**FSCGRNIKTRNKREA**N**LPDF**QTDFSDDYDEIDENNFVETPTNFSGLVPT**VQSQNATLLK
transcript/802606	E**S**YLLGEDGHSCVAGG**D**FSCGRNIKTRNKREA**N**LPDF**QTDFSDDYDEIDENNFVETPTNFSGLVPT**VQSQNATLLK
Oxyuranus microlepidotus vF10	KSDNPSPDIR**I**VNG**M**DCKLGECPWQA**V**L**VDEKEGV**FCGGTILSPIYVLTAAHCINQT**EK**I**S**VVVGEID**K**SR**VE**TG**H**
Oxyuranus microlepidotus F10	KSDNPSPDIR**V**VNG**T**DCKLGECPWQA**L**L**INDQGDG**FCGGTILSPIYVLTAAHCINQT**KY**I**R**VVVGEID**I**SR**KK**TG**R**
transcript/802606	KSDNPSPDIR**V**VNG**T**DCKLGECPWQA**L**L**INDQGDG**FCGGTILSPIYVLTAAHCINQT**KY**I**R**VVVGEID**I**SR**KK**TG**R**
Oxyuranus microlepidotus vF10	LLSVDKIYVH**K**KFVP**PKKGYKFYEKFDLVS**YDYDIAIIQ**M**KTPIQFSENVVPACLPTADFANQVLMKQ**D**FGI**I**SGF
Oxyuranus microlepidotus F10	LLSVDKIYVH**Q**KFVP-------------**AT**YDYDIAIIQ**L**KTPIQFSENVVPACLPTADFANQVLMKQ**N**FGI**V**SGF
transcript/802606	LLSVDKIYVH**Q**KFVP-------------**AT**YDYDIAIIQ**L**KTPIQFSENVVPACLPTADFANQVLMKQ**N**FGI**V**SGF
Oxyuranus microlepidotus vF10	GR**IF**E**K**G**PK**SNTLKV**LKV**PYVDRHTCM**V**SS**ES**PIT**PT**MFCAGYDTLP**R**DACQGDSGGPHITAYRDTHFITGIVSWG
Oxyuranus microlepidotus F10	GR**TR**E**R**G**QT**SNTLKV**VTL**PYVDRHTCM**L**SS**NF**PIT**QN**MFCAGYDTLP**Q**DACQGDSGGPHITAYRDTHFITGIVSWG
transcript/802606	GR**TR**E**R**G**QT**SNTLKV**VTL**PYVDRHTCM**L**SS**NF**PIT**QN**MFCAGYDTLP**Q**DACQGDSGGPHITAYRDTHFITGIVSWG
Oxyuranus microlepidotus vF10	EGCA**KK**GKYG**I**YTKVSKFILWIKRIMRQKLPSTESSTGRL
Oxyuranus microlepidotus F10	EGCA**QT**GKYG**V**YTKVSKFILWIKRIMRQKLPSTESSTGRL
transcript/802606	EGCA**QT**GKYG**V**YTKVSKFILWIKRIMRQKLPSTESSTGRL

Fig. S3. The hemostatic *F10* gene is transcribed in inland taipan (*Oxyuranus microlepidotus*) venom gland tissue. Protein sequence alignment comparing the hypothetical protein translations of the *F10* and *vF10* paralogs of *O. microlepidotus* with a representative full-length translated Iso-seq read corresponding to hemostatic factor X from venom gland tissue. The alignment provides qualitative evidence that full-length *F10* transcripts are expressed in venom gland tissue. Amino acid residues that differ between the *F10* and *vF10* sequences are shown in bold.

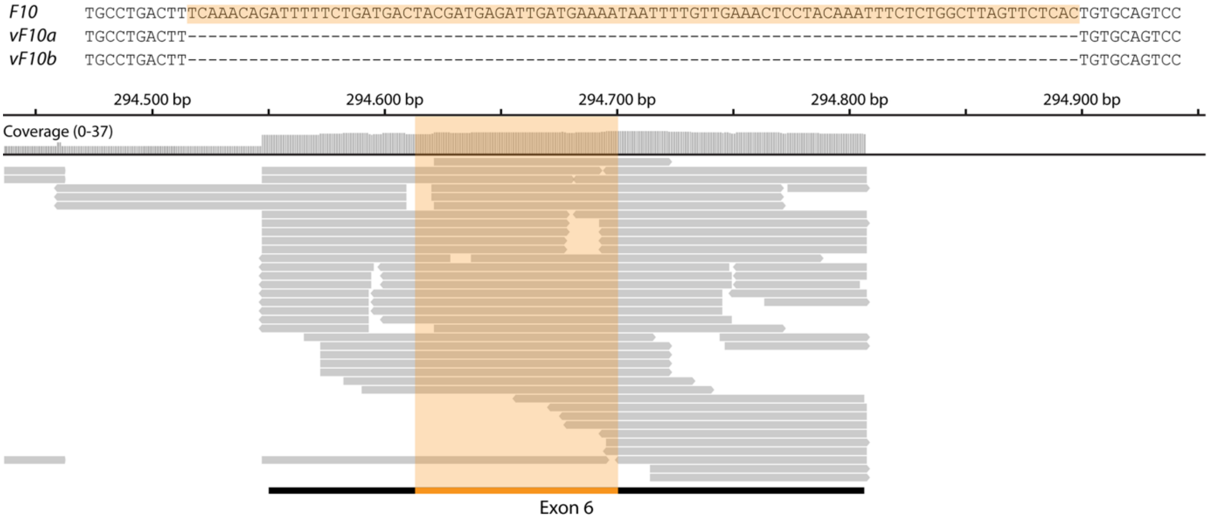

Fig. S4. The *F10* gene is expressed in Eastern brown snake (*Pseudonaja textilis*) venom gland tissue. One distinguishing feature between the *F10* and *vF10* paralogs is an 87-nucleotide deletion in exon 6 of the *vF10* gene. The corresponding nucleotide sequence in the *F10* gene (shown in orange in the sequence alignment) is absent in *vF10*. Taking advantage of this structural variant, we identified multiple short-read RNA-seq reads from *P. textilis* venom gland tissue that map to this *F10*-specific region (highlighted in orange), providing evidence that the hemostatic *F10* gene is also transcribed in the venom gland of *P. textilis*.

Pseudonaja textilis vF10a	MAPQLLLCLILTFLWSLPEAESNVFLKSKVANRFLQRTKRANSLVEEFKSGNIERECIEERCSKEEAREVFEDDEK
Pseudonaja textilis vF10b	MAPQLLLCLILTFLWSLPEAESNVFLKSKVANRFLQRTKRANSLVEEFKSGNIERECIEERCSKEEAREVFEDDEK
Pseudonaja textilis F10	MAPQLLLCLILTFLWSLPEAESNVFLKSKVANRFLQRTKRANSLFEEFKPGNIERECIEERCSKEEAREAFEDDEK
Pseudonaja textilis vF10a	TETFWNVYVDGDQCSSNPCHYRGICKDGIGSYTCTCLSGYEGKNCERVLYKSCR**VDNGNCWHFCK**SVQNDIQCSCA
Pseudonaja textilis vF10b	TETFWNVYVDGDQCSSNPCHYRGICKDGIGSYTCTCLSGYEGKNCERVLYKSCR**VDNGNCWHFCK**HVQNDIQCSCA
Pseudonaja textilis F10	TETFWNVYVDGDQCSSNPCHYGGTCKDGIGSYTCTCLSGYEGKNCEYVLYKSCR**VDNGDCWHFCK**PVQNGIQCSCA
Pseudonaja textilis vF10a	EGYLLGEDGHSCVAGGNFSCGRNIKTRNKREASLPDF-----------------------------VQSHNATLLK
Pseudonaja textilis vF10b	EGYLLGEDGHSCVAGGNFSCGRNIKTRNKREANLPDF-----------------------------VQSQNATLLK
Pseudonaja textilis F10	ESYLLGEDGHSCVAGGDFSCGRNIKTRNKREANLPDFQTDFSDDYDEIDENNFVETPTNFSGLVLTVQSQNATLLK
Pseudonaja textilis vF10a	**KSDNPSPDIRIVNGMDCK**LGECPWQAALVDDKKGVFCGGTILSPIYVLTAAHCINETETISVVVGEIDRSR**AETGP**
Pseudonaja textilis vF10b	**KSDNPSPDIRIVNGMDCK**LGECPWQAALVDEKEGVFCGGTILSPIYVLTAAHCINETETISVVVGEIDKSRIETGP
Pseudonaja textilis F10	**KSDNPSPDIR**VVNGTDCKLGECPWQALLLNDEGDGFCGGTILSPIYVLTAAHCINQTKYITVVVGEIDISSKKTGR
Pseudonaja textilis vF10a	**LLSVDKVYVHKKFVPPKKSQEFYEK**FDLVSYDYDIAIIQMKTPIQFSENVVPACLPTADFANQVLMKQDFGIVSGF
Pseudonaja textilis vF10b	LLSVDKIYVHK**KFVPPQK**AY----KFDLAAYDYDIAIIQMKTPIQFSENVVPACLPTADFANQVLMKQDFGIVSGF
Pseudonaja textilis F10	LHSVDKIYVHQKFVP-------------ATYDYDIAIIQLKTPIQFSENVVPACLPTADFANQVLMKQNFGIVSGF
Pseudonaja textilis vF10a	GGIFERGPNSKTLKVLKVPYVDRHTCMLSSNFPITPTMFCAGYDTLPQDACQGDSGGPHITAYRDTHFITGIVSWG
Pseudonaja textilis vF10b	GRIFEKGPKSKTLKVLKVPYVDRHTCMVSSETPITPNMFCAGYDTLPR**DACQGDSGGPHTTVYR**DTHFITGIVSSG
Pseudonaja textilis F10	GRTRERGKTSNTLKVVTLPYVDRHTCMLSSNFPITQNMFCAGYDTLPQDACQGDSGGPHITAYRDTHFITGIVSWG
Pseudonaja textilis vF10a	EGCARK**GRYGIYTK**LSKFIPWIKRIMR**QKLPSTESSTGRL**
Pseudonaja textilis vF10b	EGCARNGKYGIYTKLSKFIPWIKRIMR**QKLPSTESSTGRL**
Pseudonaja textilis F10	EGCAQTGKYGVYTKVSKFILWIKRIIRQKQPSTESSTGQL

Fig S5. Coagulation factor X protein is detected in Eastern brown snake (*Pseudonaja textilis*) venom. Amino acid sequence alignment of hypothetical *F10* gene translations with tryptic peptides shown in bold. Red residues indicate peptides unique to the venom paralogs (*vF10a* and/or *vF10b*), purple residues indicate peptides unique to the liver protein (*F10*), and black residues indicate peptides shared by all paralogs. Residues shown in grey correspond to the signal peptide and propeptide regions. Tryptic peptides corresponding to the hemostatic protein were detected in the venoms from three of seven individuals.

Oxyuranus microlepidotu F7b	MISCHFGVFLCLLLLASPIARTSVFLRHKEASNILQRAR**R**ANSFLEELKA**G**SIERECLE**E**KCS**W**EEAREIFQDDLR
transcript/829350	MISCHFGVFLCLLLLASPIARTSVFLRHKEASNILQRAR**R**ANSFLEELKA**G**SIERECLE**E**KCS**W**EEAREIFQDDLR
Oxyuranus microlepidotu F7a	MISCHFGVFLCLLLLASPIARTSVFLRHKEASNILQRAR**Q**ANSFLEELKA**E**SIERECLE**G**KCS**C**EEAREIFQDDLR
transcript/809936	MISCHFGVFLCLLLLASPIARTSVFLRHKEASNILQRAR**Q**ANSFLEELKA**E**SIERECLE**G**KCS**C**EEAREIFQDDLR
Oxyuranus microlepidotu F7b	TKEFW**A**IYIDPDQCDSNPCQNGGTCIDRPQDYFCICPPEHEGRNCEIGPEIKLKCF**FK**NGNCEQFCVDSPTTLRQC
transcript/829350	TKEFW**A**IYIDPDQCDSNPCQNGGTCIDRPQDYFCICPPEHEGRNCEIGPEIKLKCF**FK**NGNCEQFCVDSPTTLRQC
Oxyuranus microlepidotu F7a	TKEFW**E**IYIDPDQCDSNPCQNGGTCIDRPQDYFCICPPEHEGRNCEIGPEIKLKCF**LE**NGNCEQFCVDSPTTLRQC
transcript/809936	TKEFW**E**IYIDPDQCDSNPCQNGGTCIDRPQDYFCICPPEHEGRNCEIGPEIKLKCF**LE**NGNCEQFCVDSPTTLRQC
Oxyuranus microlepidotu F7b	SCAEGYRLASDQVSCIAEVDYPCGKIPVLAKRPNQQGRIVGGYDCPPGECPWQALITENGNEKCGGVLVAPSWIIS
transcript/829350	SCAEGYRLASDQVSCIAEVDYPCGKIPVLAKRPNQQGRIVGGYDCPPGECPWQALITENGNEKCGGVLVAPSWIIS
Oxyuranus microlepidotu F7a	SCAEGYRLASDQVSCIAEVDYPCGKIPVLAKRPNQQGRIVGGYDCPPGECPWQALITENGNEKCGGVLVAPSWIIS
transcript/809936	SCAEGYRLASDQVSCIAEVDYPCGKIPVLAKRPNQQGRIVGGYDCPPGECPWQALITENGNEKCGGVLVAPSWIIS
Oxyuranus microlepidotu F7b	AAHCFSRVRKENLRIRLGEYHLNHRDEGEQERRVDELIIHEKYSFKTVDNDIALLRLSAPVNFSEYVVPICLPPPR
transcript/829350	AAHCFSRVRKENLRIRLGEYHLNHRDEGEQERRVDELIIHEKYSFKTVDNDIALLRLSAPVNFSEYVVPICLPPPR
Oxyuranus microlepidotu F7a	AAHCFSRVRKENLRIRLGEYHLNHRDEGEQERRVDELIIHEKYSFKTVDNDIALLRLSAPVNFSEYVVPICLPPPR
transcript/809936	AAHCFSRVRKENLRIRLGEYHLNHRDEGEQERRVDELIIHEKYSFKTVDNDIALLRLSAPVNFSEYVVPICLPPPR
Oxyuranus microlepidotu F7b	FAADILNYVEFSTVSGWGRLLEGGSTSALLMRVEVPKIHKKECVRHTKFNITDNMFCAGYLDGSKDSCEGDSGGPH
transcript/829350	FAADILNYVEFSTVSGWGRLLEGGSTSALLMRVEVPKIHKKECVRHTKFNITDNMFCAGYLDGSKDSCEGDSGGPH
Oxyuranus microlepidotu F7a	FAADILNYVEFSTVSGWGRLLEGGSTSALLMRVEVPKIHKKECVRHTKFNITDNMFCAGYLDGSKDSCEGDSGGPH
transcript/809936	FAADILNYVEFSTVSGWGRLLEGGSTSALLMRVEVPKIHKKECVRHTKFNITDNMFCAGYLDGSKDSCEGDSGGPH
Oxyuranus microlepidotu F7b	VTEYKNTWYLTGLVSWGKGCAAIGTYGVYTKVIKYHQWLNNHMDS
transcript/829350	VTEYKNTWYLTGLVSWGKGCAAIGTYGVYTKVIKYHQWLNNHMDS
Oxyuranus microlepidotu F7a	VTEYKNTWYLTGLVSWGKGCAAIGTYGVYTKVIKYHQWLNNHMDS
transcript/809936	VTEYKNTWYLTGLVSWGKGCAAIGTYGVYTKVIKYHQWLNNHMDS

Fig S6. Full-length *F7* transcripts are present in inland taipan (*Oxyuranus microlepidotus*) venom gland tissue. Protein sequence alignment of the hypothetical protein translations encoded by the *F7a* and *F7b* paralogs of *O. microlepidotus*, together with representative full-length translated Iso-seq reads from venom gland tissue corresponding to each *F7* paralog. The alignment provides qualitative evidence that full-length *F7a and F7b* transcripts are expressed in venom gland tissue. Amino acid residues that differ between the *F7a* and *F7b* sequences are shown in bold.

Pseudonaja textilis F7a	MISCHFGVFLCLLLLASPIARTSVFLRHKEASNILQRARRANSFLEELKAESIEQECLEQKCSCEEAREIFQDDLR
Pseudonaja textilis F7b1	MVSCHFGVFLCLLLLASPIARTSVFLRHKEASNILQRARRANSFLEELKAGSIERECLEENCSWEEAREIFQDDMR
Pseudonaja textilis F7b2	MISWHFGVFLCLLLLASPIARTSVFLRHKEASNILQRARRANSFLEELKAGSIERECLEEKCSWEEAREIFQDDLR
Pseudonaja textilis F7a	TKEFCEIYIDPDQCDSNPCQNGGTCIDRPQDYFCICPPEHEGRNCEIGPEIKLKCFLENGNCEQFCVDSPTTLRQC
Pseudonaja textilis F7b1	TKEFWAIYIDPDQCDSNPCQNGGTCIDRPQDYFCICPPEHEGRNCEIGPETKLKCFFKNGNCEQFCVDSPTTLR**QC**
Pseudonaja textilis F7b2	TKEFWAIYIDPDQCDSNPCQNGGTCIDRPQDYFCICPPEHEGRNCEIGPETKLKCFFKNGNCEQFCVDSPTTLR**QC**
Pseudonaja textilis F7a	SCAEGYKLASDQVSCIAEVDYPCGKIPVLAKRPNQQGRIVGGYDCPPGECPWQALITENGNEKCGGVLVAPSWIIS
Pseudonaja textilis F7b1	**SCAEGYR**LASDQVSCIAEVDYPCGKIPVLAKRPNQQGRIVGGYDCPPGECPWQALITENGNEKCGGVLVAPSWVIS
Pseudonaja textilis F7b2	**SCAEGYR**LASDQVSCIAEVDYPCGKIPVLAKRPNQQGRIVGGYDCPPGECPWQALITENGNEKCGGVLVAPSWVIS
Pseudonaja textilis F7a	AAHCFSHLRKENLRIR**LGEYHLNHRDEGEQER**RVDELIIHENYSFKTVDNDIALLHLSAPVNFSEYVVPICLPPPR
Pseudonaja textilis F7b1	AAHCFSHLRKENLRIR**LGEYHLNHRDEGEQER**RVDELIIHEKYSFKTVDNDIALLRLSAPVNFSEYVVPICLPPPR
Pseudonaja textilis F7b2	AAHCFSRVHKENLRIR**LGEYHLNHRDEGEQER**RVDELIIHEKYSFKTVDNDIALLRLSAPVNFSEYVVPICLPPPR
Pseudonaja textilis F7a	FAADILNYVEFSTVSGWGRLLEGGSTSALLMRVEVPKIHKKECVRHTKFNITDNMFCAGYLDGSK**DSCEGDSGGPH**
Pseudonaja textilis F7b1	FAADVLNYVEFSTVSGWGRLLEGGSTSALLMRVQVPKIHKKECVQHTKFNITDNMFCAGYLDGSK**DSCEGDSGGPH**
Pseudonaja textilis F7b2	FAADVLNYVEFSTVSGWGRLLEGGSTSALLMRVQVPKIHKKECVRHTKFNITDNMFCAGYLDGSK**DSCEGDSGGPH**
Pseudonaja textilis F7a	**VTEYK**NTWYLTGLVSWGKGCAAIGTYGVYTKVIKYHQWLNNHMDS
Pseudonaja textilis F7b1	**VTEYK**NTWYLTGLVSWGKGCAAIGTYGVYTKVIKYHQWLNNHMDS
Pseudonaja textilis F7b2	**VTEYK**NTWYLTGLVSWGKGCAAIGTYGVYTKVIKYHQWLNNHMDS

Fig S7. Coagulation factor VII proteins are detected in Eastern brown snake (*Pseudonaja textilis*) venom. Amino acid sequence alignment of hypothetical F7 protein translations with tryptic peptides shown in bold. Residues shown in grey correspond to the signal peptide and propeptide regions. Tryptic peptides were detected in venom from three of seven individuals.

	**1** **85**
P. textilis vF5 (short)	MGRYSVSPVPKCLLLMFLGWSGLKYYQVNAAQLREYHIAAQLEDWDYNPQPEELSRLSESDLTFKKIVYREYELDFKQEKPRDAL
O. microlepidotus vF5 (short)	MGRYSVSPVPKCLLLMFLGWSGLKYYQVNAAQLREYRIAAQLEDWDYNPQPEELSRLSESELTFKKIVYREYELDFKQEKPRDEL
P. textilis F5 (short)	MGRYSVSPVPKCLLLMFLGWSGLKYYQVNAAQLREYRIAAQLEDWDYNPQPEELSRLSESDLTFKKIVYREYELDFKQEKPRDAL
O. microlepidotus F5 (short)	MGRYSVSPVPKCLLLMFLGWSGLKYYQVNAAQLREYRIAAQLEDWDYSPQPEELSRLSESELTFKKIVYREYELDFKQEKPRDEL
P. textilis F5	MGRYSVSPVPKCLLLMFLGWSGLKYYQVNAAQLREYRIAAQLEDWDYNPQPEELSRLSESDLTFKKIVYREYELDFKQEKPRDAL
O. microlepidotus F5	MGRYSVSPVPKCLLLMFLGWSGLKYYQVNAAQLREYRIAAQLEDWDYSPQPEELSRLSESELTFKKIVYREYELDFKQEKPRDEL
N. scutatus F5	MGRYSVSPVPKCLLLMFLGWSGLKYYQVNAAQLREYRIAAQLEDWDYNLQPEELSRLSESDRTFQKIVYREYELDFKQEKPRDEL
B. candidus F5	MGRYSVSPVSKCLLLMFLGWSGLKNYQVNAAQLREYRIAAQLEDWDYNLQPEGLSRLSESDRTFRKIVYREYELDFKQEKPRDKL
N. helvetica F5	MGRYSVSPIPKCLLLVFLGWSGLKYYQVNAAQLREYHIAAQVEDWDYNLQPGGLSRLSESDLAFKKIVYREYEVDFKQEKTRDEL

	**86** **170**
P. textilis vF5 (short)	SGLLGPTLRGEVGDSLIIYFKNFATQPVSIHPQSAVYNKWSEGSSYSDGTSDVERLDDAVPPGQSFKYVWNITAEIGPKKADPPC
O. microlepidotus vF5 (short)	SGLLGPTLRGEVGDILIIYFKNFATQPVSIHPQSAVYNKWSEGSSYSDGTSDVERLDDAVPPGQSFKYVWNITAEIGPKKADPPC
P. textilis F5 (short)	SGLLGPTLRGEVGDILIIYFKNFATQPVSIHPQSAVYNKWSEGSSYSDGTSDVERLDDAVPPGQSFKYVWNITAEIGPKKADPPC
O. microlepidotus F5 (short)	SGLLGPTLRGEVGDILIIYFKNFATQPVSIHPQSAVYNKWSEGSSYSDGTSDVERLDDAVPPGQSFKYVWNITAEIGPKKADPPC
P. textilis F5	SGLLGPTLRGEVGDILIIYFKNFATQPVSIHPQSAVYNKWSEGSSYSDGTSDVERLDDAVPPGQSFKYVWNITAEIGPKKADPPC
O. microlepidotus F5	SGLLGPTLRGEVGDILIIYFKNFATQPVSIHPQSAVYNKWSEGSSYSDGTSDVERLDDAVPPGQSFKYVWNITAEIGPKKADPPC
N. scutatus F5	SGLLGPTLRGEVGDILIIYFKNFATKPVSIHPQSAVYNKWSEGSSYSDGTSDVERLDDAVPPGQSFKYVWNITAEIGPKKADPPC
B. candidus F5	SGLLGPTLRGEVGDILIIYFKNFATQPVSIHPQSAVYNKWSEGSSYSDGTSDMERLDNAVPPGQAFKYVWNITAEIGPKKADPPC
N. helvetica F5	SGLLGPTLRGEVGDVLVIYFKNFATQPVSIHPQSAVYNKWSEGASYSDGTSDVEKLDDAVPPDHSFKYVWNITAEIGPKETDPPC

	**171** **255**
P. textilis vF5 (short)	LTYAYYSHVNMVRDFNSGLIGALLICKEGSLNANGSQKFFNREYVLMFSVFDESKNWYRKPSLQYTINGFANGTLPDVQACAYDH
O. microlepidotus vF5 (short)	LTYAYYSHVNMVRDFNSGLIGALLICKEGSLNANGAQKFFNREYVLMFSVFDESKNWYRKPSLQYTINGFANGTLPDVQACAYDH
P. textilis F5 (short)	LTYAYYSHVNMVRDFNSGLIGALLICKEGSLNANGAQKFFNREYVLMFSVFDESKNWYRKPSLQYTINGFANGTLPDVQACAYDH
O. microlepidotus F5 (short)	LTYSYYSHVNMVRDFNSGLIGALLICKEGSLNANGAQKFFNREYVLMFSVFDESKNWYRKPSLQYTINGFANGTLPDVQACAYDH
P. textilis F5	LTYAYYSHVNMVRDFNSGLIGALLICKEGSLNANGAQKFFNREYVLMFSVFDESKNWYRKPSLQYTINGFANGTLPDVQACAYDH
O. microlepidotus F5	LTYSYYSHVNMVRDFNSGLIGALLICKEGSLNANGAQKFFNREYVLMFSVFDESKNWYRKPSLQYTINGFANGTLPDVQACAYDH
N. scutatus F5	LTYAYYSHVNMVRDFNSGLIGALLICKEGSLNANGTQKLFNREYVLMFSVFDESKNWYRKPSLQYTINGFANGTLPDVQACAYDH
B. candidus F5	LTYAYYSHVNMVQDFNSGLIGALLICKKGSLTANGTQKLFNREYVLMFSVFDESKNWYKKPSLQYTINGFANGTLPDIQACAYDR
N. helvetica F5	LTYAYYSHVNMVRDFNSGLIGALLICKEGSLNADGTQKLFNREYVLMFAVFDESKNWYKKPSLQYTINGFANGTLPDIQACAYDR

	**256** **340**
P. textilis vF5 (short)	ISWHLIGMSSSPEIFSVHFNGQTLEQNHYKVSTINLVGGASVTADMSVSRTGKWLISSLVAKHLQAGMYGYLNIKDCGNPDTLTR
O. microlepidotus vF5 (short)	ISWHLIGMSSSPEIFSVHFNGQTLEQNHYKVSTINLVGGASVTANMSVSRTGKWLISSLVAKHLQAGMYGYLNIKDCGHPNTLTR
P. textilis F5 (short)	ISWHLIGMSSSPEIFSVHFNGQTLEQNHYKVSTINLVGGASVTANMSVSRTGKWLISSLVAKHLQAGMYGYLNIKDCGNPDTLTR
O. microlepidotus F5 (short)	ISWHLIGMSSSPEIFSVHFNGQTLEQNHYKVSTINLVGGASVTANMSVSRTGKWLISSLVAKHLQAGMYGYLNIKDCGNPDTLTR
P. textilis F5	ISWHLIGMSSSPEIFSVHFNGQTLEQNHYKVSTINLVGGASVTANMSVSRTGKWLISSLVAKHLQAGMYGYLNIKDCGNPDTLTR
O. microlepidotus F5	ISWHLIGMSSSPEIFSVHFNGQTLEQNHYKVSTINLVGGASVTANMSVSRTGKWLISSLVAKHLQAGMYGYLNIKDCGNPDTLTR
N. scutatus F5	ISWHLIGMSSSPEIFSVHFNGQTLEQNHYKVSTINLIGGASVTAKMSVSRTGKWLISSLVAKHLQAGMYGYLNIKDCGNPDTLTR
B. candidus F5	ISWHLIGMSSSPEIFSVHFNGQTLEQNNYKVSTINLVGGASVTANMSVSRTGKWLISSLVAKHLQAGMYGYLNIKDCGKSDTLTR
N. helvetica F5	ISWHLIGMSSSPEIFSVHFNGQTLEENHYKVSTINLVGGASVTANMSVSMTGKWLISSLVAKHLQAGMYGYLNIKDCGKPDTLTK

	**341** **425**
P. textilis vF5 (short)	KLSFRELMKIKNWEYFIAAEEITWDYAPEIPSSVDRRYKAQYLDNFSNFIGKKYKKAVFRQYEDGNFTKPTYAIWPKERGILGPV
O. microlepidotus vF5 (short)	KLSFRELRRIMNWEYFIAAEEITWDYAPEIPSSVDRRYKAQYLDNFSNFIGKKYKKAVFRQYEDGNFTKPTYAIWPKERGILGPV
P. textilis F5 (short)	KLSFRELRRIMNWEYFIAAEEITWDYAPEIPSSVDRRYKAQYLDNFSNFIGKKYKKAVFRQYKDSNFTKPTYAIWPKERGILGPV
O. microlepidotus F5 (short)	KLSFRELRRIMNWEYFIAAEEITWDYAPEIPSSVDRRYKAQYLDNFSNFIGKKYKKAVFRQYKDGNFTKPTYAIWPKERGILGPV
P. textilis F5	KLSFRELRRIMNWEYFIAAEEITWDYAPEIPSSVDRRYKAQYLDNFSNFIGKKYKKAVFRQYKDSNFTKPTYAIWPKERGILGPV
O. microlepidotus F5	KLSFRELRRIMNWEYFIAAEEITWDYAPEIPSSVDRRYKAQYLDNFSNFIGKKYKKAVFRQYKDGNFTKPTYAIWPKERGILGPV
N. scutatus F5	KLSFRELKMIKNWEYFIAAEEITWDYAPEIPRSVDRRYKAQYLDNFSNFIGKKYKKAVFRQYKDSSFSKPTYAIWPKERGILGPV
B. candidus F5	KLSFKELRMIKNWEYFIAAEEITWDYAPEIPSTVDRRYKAQYLDNFSNFIGKKYKKAVFRQYKDSSFTKPTYAIWPKERGILGPV
N. helvetica F5	RLTFKELRMIKNWDYFIAAEEITWDYAPEIPSSVDRRYKAQYLDNFSNFIGKKYKKAVFRQYKDSSFSKPTYATWPKERGILGPV

	**426** **510**
P. textilis vF5 (short)	IKAKVRDTVTIVFKNLASRPYSIYVHGVSVSKDAEGAIYPSDPKENITHGKAVEPGQVYTYKWTVLDTDEPTVKDSECITKLYHS
O. microlepidotus vF5 (short)	IKAKVRDTVTIVFKNLASRPYSIYVHGVSVSKDAEGAIYPSDPKENITHGKAVEPGQVYTYKWTVLDTDEPTVKDSECITKLYHS
P. textilis F5 (short)	IRAKVRDTISIVFKNLASRPYSIYVHGVSVSKDAEGAIYPSDPKENITHGKAVEPGQVYTYKWTVLDTDEPTVKDSECITKLYHS
O. microlepidotus F5 (short)	IRAKVRDTISIVFKNLASRPYSIYVHGVSVSKDAEGAIYPSDPKENITHGKAVEPGQVYTYKWTVLDTDEPTVKDSECITKLYHS
P. textilis F5	IRAKVRDTISIVFKNLASRPYSIYVHGVSVSKDAEGAIYPSDPKENITHGKAVEPGQVYTYKWTVLDTDEPTVKDSECITKLYHS
O. microlepidotus F5	IRAKVRDTISIVFKNLASRPYSIYVHGVSVSKDAEGAIYPSDPKENITHGKAVEPGQVYTYKWTVLDTDEPTVKDSECITKLYHS
N. scutatus F5	IRAEVRDTISIVFKNLASRPYSIYVHGVSVSKDAEGAIYPSDPKENITHGKAVEPGQVYTYKWTVLDTDEPTVKDSQCITKLYHS
B. candidus F5	IRAEVRDTISIVFKNLASRPYSIYVHGVSVSKDAEGAIYPSDPKENITHGKAVEPGHIYTYKWTVLDTDEPTVKDSQCITKLYHS
N. helvetica F5	IRAGVRDTISIVFKNLASRPYSIYVHGVSVSKDAEGAVYPSDSKENITQGKAVEPGDVYTYKWTVLDTDEPTAKDSQCITKLYHS

	**511** **595**
P. textilis vF5 (short)	AVDMTRDIASGLIGPLLVCKHKALSVKGVQNKADVEQHAVFAVFDENKSWYLEDNIKKYCSNPSAVKKDDPKFYKSNVMYTLNGY
O. microlepidotus vF5 (short)	AVDMTRDIASGLIGPLLVCKLKALSVKGVQNKADVEQHAVFAVFDENKSWYLEDNIKKYCSNPSSVKKDDPKFYKSNVMYTLNGY
P. textilis F5 (short)	AVDMTRDIASGLIGPLLVCKHKALSVKGVQNKADVEQHAVFAVFDENKSWYLEDNIKKYCSNPSTVKKDDPKFYKSNVMYTLNGY
O. microlepidotus F5 (short)	AVDMTRDIASGLIGPLLVCKRKALSVKGVQNKADVEQHAVFAVFDENKSWYLEDNIKKYCSNPSSVKKDDPKFYKSNVMYTLNGY
P. textilis F5	AVDMTRDIASGLIGPLLVCKHKALSVKGVQNKADVEQHAVFAVFDENKSWYLEDNIKKYCSNPSTVKKDDPKFYKSNVMYTLNGY
O. microlepidotus F5	AVDMTRDIASGLIGPLLVCKRKALSVKGVQNKADVEQHAVFAVFDENKSWYLEDNIKKYCSNPSSVKKDDPKFYKSNVMYTLNGY
N. scutatus F5	AVDMTRDIASGLIGPLLVCKRKALNSKGVQDKADVEQHAVFAVFDENKSWYLEDNIKKYCSNSSTVKKDDPKFYKSNVMYTLNGY
B. candidus F5	AVDMTRDIASGLIGPLLVCKRKALDSKGVQKKADVEQHAVFAVFDENKSWYLEDNIKKYCSNSSTVKKDDPKFYKSNVMYTLNGY
N. helvetica F5	AVDMTRDIASGLIGPLLVCKRKALDNRGVQKKADVEQHAIFAVFDENKSWYLDDNIKKYSSNFSTVKKDDPKFYKSNVMYTLNGY

596 — 680

P. textilis vF5 (short)	ASDRTEVLRFHQSEVVQWHLTSVGTVDEIVPVHLSGHTFLSKGKHQDILNLFPMSGESATVTMDNLGTWLLSSWGSCEMSNGMRL
O. microlepidotus vF5 (short)	ASDRTEVLGFHQSEVVQWHLTSVGTVDEIVPVHLSGHTFLSKGKHQDILNLFPMSGESATVTMDNLGTWLLSSWGSCEMSNGMRL
P. textilis F5 (short)	ASDRTEVLGFHQSEVVEWHLTSVGTVDEIVPVHLSGHTFLSKGKHQDILNLFPMSGESATVTMDNLGTWLLSSWGSCEMSNGMRL
O. microlepidotus F5 (short)	ASDRTEVLGFHQSEVVEWHLTSVGTVDEIVPVHLSGHTFLSKGKHQDILNLFPMSGESATVTMDNLGTWLLSSWGSCEMSNGMRL
P. textilis F5	ASDRTEVLGFHQSEVVEWHLTSVGTVDEIVPVHLSGHTFLSKGKHQDILNLFPMSGESATVTMDNLGTWLLSSWGSCEMSNGMRL
O. microlepidotus F5	ASDRTEVLGFHQSEVVEWHLTSVGTVDEIVPVHLSGHTFLSKGKHQDILNLFPMSGESATVTMDNLGTWLLSSWGSCEMSNGMRL
N. scutatus F5	ASDRTDVLGFRQSEIVEWHLTSVGTVDEIVPVHLSGHTFLSKGKHQDILNLFPMSGESATVTMDNLGTWLLSSWGSREMSNGMRL
B. candidus F5	ASDRTDVLGFRQSEIVEWHLTSVGTVDEIVPVHLSGHTFLSKGKHQDILNLFPMRGESATVTMDNLGTWLLSSWGSREMSNGMRL
N. helvetica F5	ASDRTEVLGFHQSEIVEWHLTSVGTMDEIIPVHLSGHTFLSKGKHQDILNLFPMSGESATVTMDNLGTWLLSSWGSREMSNGMRL

681 — 765

P. textilis vF5 (short)	RFLDANYDDEDE**GNEEEEEDDGDIFADI--FIPSEVVKK---KEEVPVNFVPDPESDALAKELGLIDDEGNPIIQPRREQTEDDE**
O. microlepidotus vF5 (short)	RFLDANYDDEDE**GNEEEEEDDGDIFADI--FSPPEVVKK---KEEVPVNFVPDPESDALAKELGLLDDEDNP-EQSRSEQTEDDE**
P. textilis F5 (short)	RFLDANYDDEDE**GNEEEEEDDGDIFADI--FIPPEVVKK---KEKDPVNFVSDPESDKIAKELGLLDDEDNQ-EESHNVQTEDDE**
O. microlepidotus F5 (short)	RFLDANYDDEDE**GNEEEEEDDGDIFADI--FSPPEVVKK---KEKDPVNFVSDPESDKIAKELGLLDDEDNQ-EESHNEQTEDDE**
P. textilis F5	RFLDANYDDEDE**GNEEEEEDDGDIFADI--FIPPEVVKK---KEKDPVNFVSDPESDKIAKELGLLDDEDNQ-EESHNVQTEDDE**
O. microlepidotus F5	RFLDANYDDEDE**GNEEEEEDDGDIFADI--FSPPEVVKK---KEKDPVNFVSDPESDKIAKELGLLDDEDNQ-EESHNEQTEDDE**
N. scutatus F5	RFLDAKHDDEDE**GNEKEEEHYGGIVANIISGGPSEVVKK---KEKDPRNVVSDSEMDKIAKELGLLDDEDNQ-KESSNEQTEDDE**
B. candidus F5	RFLDANYDDEDE**GNEKEEEEDGDIVANIVSFSSPDVIKE---KRKDSKSVVSDPETDKIAKELGLLDDEDNQ-KESSNEQIEDDE**
N. helvetica F5	RFLDANYDDEDD**RDEK-EEDYDDIVANIVSFDPSEVTEKKEEKEKDLRKVVSDPESDEIAKQLGLLDDEDST-YKSSNEQAEDDE**

766 — 850

P. textilis vF5 (short)	**EQLMKASMLGLRSFKGSVA--EEELKHTALALEEDAHAS--**
O. microlepidotus vF5 (short)	**EQLMIASMLGLRSFKGSVA--EEELKHTALALEEDAHAS--**
P. textilis F5 (short)	**EQLMIATMLGFRSFKGSVA--EEELNLTALALEEDAHAS--**
O. microlepidotus F5 (short)	**EQLMIATMLGLRSFKGSVA--EEELNLTALALEEDAHAS--**
P. textilis F5	**EQLMIATMLGFRSFKGSVA--EEELNLTALALEEDAHASGELSLNTHLVLINSTDAMRGNEELGTQNNSYVNASETIYANYTSST**
O. microlepidotus F5	**EQLMIATMLGLRSFKGSVA--EEELNLTALALEEDAHASGELSLNTHLVLINSTDAMRGNEELGTRNNSYVNASETIYANYTSST**
N. scutatus F5	**EQLMIASMLGLRSFNGSVA--EEELNLTALALEEDAHTSGELSLNTHLVLINSTDAMRGNEELGTQNNSYVNASETIYANYTSST**
B. candidus F5	**EQLMIASMLGLRTFNGSVA-EEEELNLTALALQEDAHTSGDVSLNTHLVLINSTDAMHGNEELGTQNNSYVTASETIYANYTSST**
N. helvetica F5	**EQLMIASMLGLRSFNGSVAEEEEELNLTALAIQEDADASGDVSLNDYIALNNSSDAMLGNGELGTQNNSYVTASETIYANCTSLA**

851 — 935

P. textilis vF5 (short)	**---**
O. microlepidotus vF5 (short)	**---**
P. textilis F5 (short)	**---**
O. microlepidotus F5 (short)	**---**
P. textilis F5	**GEILISNITAMIGILLSKRSQNEENITQISDSYKEMLAAEYLKAGRGLQSKDIEDLMSPEIIPSLKEQEENTFEKDIWDKKGERQ**
O. microlepidotus F5	**GEILISNITAMIGILLSKKSQNEENITQISDSYKQMLAAEYLKAGRGLQSQDIEDLMSPEIIPSLKEQEENTFEKDIWDKKGERQ**
N. scutatus F5	**GEILISNITAMIGILLSKKSQNEENITQISDSYKQMLAAEYLKAGRGLQSKDIEDLMSPEIIPSLKEQEENTFEKDIWDKQGERQ**
B. candidus F5	**GEILISNITEMIGILLSKKSQNEENITQILDSYKQMLAAEYLKAGRGLQSKDIEDLISLEIIPSLKKQEENTFEKDIWDKKGERQ**
N. helvetica F5	**GEILTSNITAVIDILLSKISQNEENITQISDSCKQFLAAEYLKTDRGPQSKDIEDLMSLEIISSMKEQEENTFEKYIWDKKRETQ**

936 — 1020

P. textilis vF5 (short)	**---**
O. microlepidotus vF5 (short)	**---**
P. textilis F5 (short)	**---**
O. microlepidotus F5 (short)	**---**
P. textilis F5	**ILTEATLHALKEMHALFFYVQQKRNLSAFNITSEGMKFLPSTENDSTIDNSKANYFSYDYDYDYKEEEATAAANVEFSKVIINTI**
O. microlepidotus F5	**ILTEATLHALKEMHALFFYVQQKRNLSAFNITSEDMKFLPSTENDSTIDNSKANYFSYDYDYDYKEEEATAAANVEFSKVIINTI**
N. scutatus F5	**ILTEATLHALKEMHALFFYVQQKRNLSAFNITSEDMKFLSSTENDSTTDNSKANYFSYDYDYDYKEEEATAAANVEFSKVITNTI**
B. candidus F5	**VLSEATLHALKEMYALFFYVQQKRNISAFNITNEDMKFLSSTENDSTLDNSKANYFSYDYDYDYKGGEATAAANVEFPKVITNAT**
N. helvetica F5	**VLTETTLYALKEMYALFFHVQQKRNLSAFNITSEDMKFLPSTENDSTLDDSKANYFPYEYDYDYK-EEATAAANVEFSKVIMNIT**

1021 — 1105

P. textilis vF5 (short)	**---**
O. microlepidotus vF5 (short)	**---**
P. textilis F5 (short)	**---**
O. microlepidotus F5 (short)	**---**
P. textilis F5	**SDDKKTVENNRSEVNLVSVPSIMPQPENLTSDATLDNFVSTTISKTTKTWNSSSHQRQKCLPKNTDMEW----KKENYDTLADIP**
O. microlepidotus F5	**SDDKKTVENNRSEVNLVSVPSIMPQPENLTSDATLDNFVSTTISKTTKAWNSSSHQRQKCLPKNTDMEW----KKENYDTLADIP**
N. scutatus F5	**SDDKKTVENNGSEVNLASVPSIMPQPENLTSDATLDNFVSTTILKTTKTWNSSSHQRQKCLPKNTDMEW----KKENYDTLADIT**
B. candidus F5	**SADKKTVENNGSELNLVSVPSIMPQPENLTSDATLDNFVSTTISKTTKTWNSSSHQKQKCLPKNTGMGW----KKENYDTLADIT**
N. helvetica F5	**SADKETVENNGSEPNLVSVPSIMPQSENLTSNATLDNFVSTTISNSTKTWKRSSPQRQKCFPKNTDKEWNVVDKKENYDILADIT**

1106 — 1190

P. textilis vF5 (short)	**---**
O. microlepidotus vF5 (short)	**---**
P. textilis F5 (short)	**---**
O. microlepidotus F5 (short)	**---**
P. textilis F5	**NDVDNKFQNSSRNASFYQENAMFPGKRKEKTDNSNGQLELGQPGITNKHDSQKDENCTINISQSFIKIQRKKKKPKVLTPRTSKH**
O. microlepidotus F5	**NDVDNKFQNSSRNASFYQENAMFPGKRKEKTDNSNGQLELGQPGITNKHDSQKDENCTINISQSFIKIQRKKKKPKVLTPRTSKH**
N. scutatus F5	**NDVDNKFQNSSRNASFYQENAMFPGKRKEKTDNSNGQLELGQPGITNKHDSQKDENCTINISQSFIKIQRKKKKPKVLTPRASKH**
B. candidus F5	**NDGDNKFQNSSRNASFYQENAMFPDKRKEKTDNSNGQLELGQPGITNKHDAQKDENCTINISQSFIKIQRKKKKPKVLTPRTSKP**
N. helvetica F5	**NDVDNKFQNSSRNASFYQENALFPGKRKEKTDNSNGQLELGQPGINNKNDAQKDENCTINISQPFVKIQRKKKKPNVLAPRTFKP**

	1191 … 1275
P. textilis vF5 (short)	**--DPRIDSN**
O. microlepidotus vF5 (short)	**--DPRIDSN**
P. textilis F5 (short)	**--DPRIDSN**
O. microlepidotus F5 (short)	**--DPRIDSN**
P. textilis F5	**LELPRNLNNSQLSNKPNHTKLLNEAKLTRKQDKLIVTIGLPVEDGDYQEYDIDNTDNDRSSSGSFEYELMYYNNPYTTDPRIDSN**
O. microlepidotus F5	**LELPRNLNNSQLSNKPNHTKLLNEAKLTRKQDKFIVTIGLPVEDGDYQEYEIDNTDNDKSSSGSFEYELMYYNNPYTTDPRIDSN**
N. scutatus F5	**LELPRNLNHSQLSNKPNHTKLLNEAKLTRKQDK-LVTIGLPVEDGDYQEYEIDNTDNDKSSSGSFEYELMYYDNPYTTDPRIDSN**
B. candidus F5	**LDLPRNLNNSQFSNKPNHTKLLNEAKLTRKQDKPIVTIGLPVEDGDYQEYEIDNIDNDQSSSGSFEYELMFYDNPYTTDSRLDST**
N. helvetica F5	**LELPANLNNSQLSNKLNHTKLLNEAKLTQKQDKPIITIGLPVEDGGYQEYEIDNTDNDESSSGSFEYELMYYDNPYTTDSRLDSN**

	1276 … 1360
P. textilis vF5 (short)	**SARNPDDIAGRYLR**TINRGNKRRYYIAAEEVLWDYSPIGKSQVRSRAAKTTFKKAIFRSYLDDTFQTPSTGGEYEKHLGILGPII
O. microlepidotus vF5 (short)	**SARNSDDIAGRYLR**TINRRNKRRYYIAAEEVLWDYSPIGKSQVRSLPAKTTFKKAIFRSYLDDTFQTPSTGGEYEKHLGILGPII
P. textilis F5 (short)	**SARNPDDIAGRYLR**TINRGNKRRYYIAAEEVLWDYSPIGKSQVRSRAAKTTFKKAIFRSYLDDTFQTPSTGGEYEKHLGILGPII
O. microlepidotus F5 (short)	**SARNPDDIAGRYLR**TINRGNKRRYYIAAEEVLWDYSPIGKSQVRSRAAKTTFKKAIFRSYLDDTFQTPSTGGEYEKHLGILGPII
P. textilis F5	**SARNPDDIAGRYLR**TINRGNKRRYYIAAEEVLWDYSPIGKSQVRSRAAKTTFKKAIFRSYLDDTFQTPSTGGEYEKHLGILGPII
O. microlepidotus F5	**SARNPDDIAGRYLR**TINRGNKRRYYIAAEEVLWDYSPIGKSQVRSRAAKTTFKKAIFRSYLDDTFQTPSTGGEYEKHLGILGPII
N. scutatus F5	**SARNPDDIARHYLR**TTNSGNKRMYYIAAEEVLWDYSPIRKSQVRSHPAKTTFKKAIFRSYLDDTFQTPSTGGEYEKHLGILGPII
B. candidus F5	**SARNPDDIAGRYMR**TINRGNIRKYYIAAEEVLWDYSPIRKSQVRSRPVKTTFKKAIFRSYLDDTFQTPSTGGEYEKHLGILGPII
N. helvetica F5	**IARNPDDIAGRYLR**TINRGNIRRYYIAAEEIVWDYSPFRKSTVRSRPLRTTFKKAIFRSYLDDTFQTPSPVGEYEKHLGILGPII

	1361 … 1445
P. textilis vF5 (short)	RAEVDDVIEIQFRNLASRPYSLHAHGLLYEKSSEGRSYDDKSPELFKKDDAIMPNGTYTYVWQVPPRSGPTDNTEKCKSWAYYSG
O. microlepidotus vF5 (short)	RAEVDDVIEVQFRNLASRPYSLHAHGLLYEKSSEGRSYDDNSPELFKKDDAIMPNGTYTYVWQVPPRSGPTDNTEKCKSWAYYSG
P. textilis F5 (short)	RAEVDDVIEVQFRNLASRPYSLHAHGLLYEKSSEGRSYDDKSPELFKKDDAIMPNGTYTYVWQVPPRSGPTDNTEKCKSWAYYSG
O. microlepidotus F5 (short)	RAEVDDVIEVQFRNLASRPYSLHAHGLLYEKSSEGRSYDDKSPELFKKDDAIMPNGTYTYVWQVPPRSGPTDNTEKCKSWAYYSG
P. textilis F5	RAEVDDVIEVQFRNLASRPYSLHAHGLLYEKSSEGRSYDDKSPELFKKDDAIMPNGTYTYVWQVPPRSGPTDNTEKCKSWAYYSG
O. microlepidotus F5	RAEVDDVIEVQFRNLASRPYSLHAHGLLYEKSSEGRSYDDKSPELFKKDDAIMPNGTYTYVWQVPPRSGPTDNTEKCKSWAYYSG
N. scutatus F5	RAEVDDVIEVQFKNLASRPYSLHAHGLLYDKSSEGRSYDDQSPELFKKDEAIMPNGTYTYVWHVPQRSGPTDNTEKCKSWAYYSG
B. candidus F5	RAEVDDVIQVQFRNLASRPYSLHAHGLLYEKSSEGRSYDDQSPEFFKMDDAIMPNGTYTYVWQVTKRSGPTDNTEKCKSWAYYSG
N. helvetica F5	RAEVDDVIQVQFRNLASRPYSLHAHGLLYEKSSEGRSYDDHSPEFFKKDDAIMPNSTYTYVWQVTKRAGPTDDTEKCKTWAYYSG

	1446 … 1530
P. textilis vF5 (short)	VNPEKDIHSGLIGPILICQKGMIDKYNRTIDIREFVLFFMVFDEEKSWYFPKSDKSTCEEKLIGVQSLHTFPAINGIPYQLQGLT
O. microlepidotus vF5 (short)	VNPEKDIHSGLIGPILICQKGMIDKYNRTIDIREFVLFFMVFDEEKSWYFPKSDKSTCEEKLIGVQSHHTFPAINGIPYQLQGLM
P. textilis F5 (short)	VNPEKDIHSGLIGPILICQKGMIDKYNRTIDIREFVLFFMVFDEEKSWYFPKSDKSTRAEKLIGVQSRHTFPAINGIPYQLQGLT
O. microlepidotus F5 (short)	VNPEKDIHSGLIGPILICQKGMIDKYNRTIDIREFVLFFMVFDEEKSWYFPKSDKSTRAEKLIGVQSRHTFPAINGIPYQLQGLM
P. textilis F5	VNPEKDIHSGLIGPILICQKGMIDKYNRTIDIREFVLFFMVFDEEKSWYFPKSDKSTRAEKLIGVQSRHTFPAINGIPYQLQGLT
O. microlepidotus F5	VNPEKDIHSGLIGPILICQKGMIDKYNRTIDIREFVLFFMVFDEEKSWYFPKSDKSTRAEKLIGVQSRHTFPAINGIPYQLQGLM
N. scutatus F5	VNPEKDIHSGLIGPILICQKGMIDKYNKTIDIREFVLFFMVFDEERSWYFAKSDKRTRAEKLIGVQSRHTFPAINGIPYQLQGLK
B. candidus F5	VNPEKDIHSGLIGPILICQKGMIDEYNRPIDIREFVLFFMVFDEERSWYFAKSDKRTRAEKLIGVQSRHTFPAINGIPYQLQGLK
N. helvetica F5	VNPEKDIHSGLIGPILICHKHMIDEYNRPIDIREFVLLFMVFDEERSWYFEKSDKRTRTERLLGVQSRHTFPAINGIPYQLQGLK

	1531 … 1615
P. textilis vF5 (short)	MYKDENVHWHLLNMGGPKDIHVVNFHGQTFTEEGREDNQLGVLPLLPGTFASIKMKPSKIGTWLLETEVGENQERGMQALFTVID
O. microlepidotus vF5 (short)	MYKDENVHWHLLNMGGPKDIHVVNFHGQTFTEEGREDNQLGVLPLLPGTFASIKMKPSKIGTWLLETEVGENQERGMQALFTVID
P. textilis F5 (short)	MYKDENVHWHLLNMGGPKDIHVVNFHGQTFTEEGREDNQLGVLPLLPGTFASIKMKPSKIGTWLLETEVGENQERGMQALFTVID
O. microlepidotus F5 (short)	MYKDENVHWHLLNMGGPKDIHVVNFHGQTFTEEGREDNQLGVLPLLPGTFTSIKMKPSKIGTWLLETEVGENQERGMQALFTVID
P. textilis F5	MYKDENVHWHLLNMGGPKDIHVVNFHGQTFTEEGREDNQLGVLPLLPGTFASIKMKPSKIGTWLLETEVGENQERGMQALFTVID
O. microlepidotus F5	MYKDENVHWHLLNMGGPKDIHVVNFHGQTFTEEGREDNQLGVLPLLPGTFTSIKMKPSKIGTWLLETEVGENQERGMQALFTVID
N. scutatus F5	MYKDENVHWHLLNMGGPKDIHVVKFHGQTFTEEGMEDNQLGVLPLLPGTFTSIKMKPSKIGTWLLETEVGENRERGMQALFTVID
B. candidus F5	MYKDENVHWHLLNMGGPKDIHVVNFHGQTFTEEGREDNQLGVLPLLPGTFTSIKMKPSKIGTWLLETEVGEYQERGMQALFTVID
N. helvetica F5	MYKEENVHWHLLNMGGPKDIHVVHFHGQTFTEEGREDNQLGVLPLLPGTFTTIKMKPSKIGTWLLKTEVGEYQERGMKALFTVID

	1616 … 1700
P. textilis vF5 (short)	KDCKLPMGLASGIIQDSQISASGHVGYWEPKLARLNNTAIFNAWSIIKKEHEHPWIQIDLQRQVVITGIQTQGTVQLLQHSYTVE
O. microlepidotus vF5 (short)	KDCKLPMGLASGIIQDSQISASGHVEYWEPKLARLNNTGMFNAWSIIKKEHEHPWIQIDLQRQVVITGIQTQGTVQLLKHSYTVE
P. textilis F5 (short)	KDCKLPMGLASGIIQDSQISASGHVGYWEPKLARLNNTGKYNAWSIIKKEHEHPWIQIDLQRQVVITGIQTQGAMQLLKHLYTVE
O. microlepidotus F5 (short)	KDCKLPMGLASGIIQDSQISASGHVGYWEPKLARLNNPGKYNAWSIIKKEHELPWIQIDLQRQVVITGIQTQGAMQLLKHLYTVE
P. textilis F5	KDCKLPMGLASGIIQDSQISASGHVGYWEPKLARLNNTGKYNAWSIIKKEHEHPWIQIDLQRQVVITGIQTQGAMQLLKHLYTVE
O. microlepidotus F5	KDCKLPMGLASGIIQDSQISASGHVGYWEPKLARLNNPGKYNAWSIIKKEHELPWIQIDLQRQVVITGIQTQGAMQLLKHLYTVE
N. scutatus F5	KDCKLPMGLASGIIQDSQISASGHVGYWEPKLARLNNPGKYNAWSIIKKEHEHPWIQIDLQRQVVITGIQTQGAMQLLKHLYTVE
B. candidus F5	KDCKLPMGLASGIIQDSQITASGHVGYWEPKLARLNNTGKYNAWSIIKREHEHPWIQVDLQRQVVITGIQTQGAMQLLKHLYTVE
N. helvetica F5	KDCKLPMGLESGIIQDSQIIASDHLGFWEPKLARLNNAGKYNAWSTIKKENEHPWIQVDLQREVVITGIQTQGAMQLVKHLYTVE

	1701 … 1785
P. textilis vF5 (short)	YFVTYSEDGQNWITFKGRHSETQMHFEGNSDGTTVKENHIDPPIIARYIRLHPTKFYNRPTFRIELLGCEVEGCSVPLGMESGAI
O. microlepidotus vF5 (short)	YFVTYSKDGQNWITFKGRHSETQMHFEGNSDGTTVKENHIDPPIIARYIRLHPTKFYNTPTFRIELLGCEVEGCSVPLGMESGAI
P. textilis F5 (short)	YFFTYSKDGQNWITFKGRHSETQMHFEGNSDGTTVKENHIDPPIIARYIRLHPTKFHNRPTFRIELLGCEVEGCSVPLGMESGAI
O. microlepidotus F5 (short)	YFVTYSKDGQNWITFKGRHSETQMHFEGNSDGTTVKENHTDPPIIARYIRLHPTKFHNRPTFRIELLGCEVEGCSVPLGMESGAI
P. textilis F5	YFFTYSKDGQNWITFKGRHSETQMHFEGNSDGTTVKENHIDPPIIARYIRLHPTKFHNRPTFRIELLGCEVEGCSVPLGMESGAI
O. microlepidotus F5	YFVTYSKDGQNWITFKGRHSETQMHFEGNSDGTTVKENHTDPPIIARYIRLHPTKFHNRPTFRIELLGCEVEGCSVPLGMESGAI
N. scutatus F5	YFVTYSKDGQNWITFKGRHSETQMHFEGNSDGSTVKENHIDPPIIARYIRLHPTKFHNRPTFRIELLGCEVEGCSVPLGMESGAI
B. candidus F5	YFVTYSKDGQKWITFKGRHSKTQMHFKGNSDGTKVKENHIDPPIIARYIRLHPTKFYNRPTFRIELLGCEVEGCSVPLGMESGAI
N. helvetica F5	YCVAYSKDGRKWITFKGRHPKTEMHFEGNSDGTKVKENHIDPPIIARYIRLHPTKFYNRPTFRIELLGCEVEGCSMPLGMESGAI

	1786 … 1870
P. textilis vF5 (short)	KNSEITASSYKKTWWSSWEPFLARLNLEGGTNAWQPEVNNKDQWLQIDLQHLTKITSIITQGATSMTTSMYVKTFSIHYTDDNST
O. microlepidotus vF5 (short)	KNSEITASSYKKTWWSSWEPFLARLNLEGGTNAWQPEVNNKDQWLQIDLQHLTKITSIITQGATSMTTAMYVKTFSIHYTDDNST
P. textilis F5 (short)	KNSEITASSYKKTWWSSWEPSLARLNLKGRTNAWQPKVNNKDQWLQIDLQHLTKITSIITQGATSMTTSMYVKTFSIHYTDDNST
O. microlepidotus F5 (short)	KDSEITASSYKKTWWSSWEPFLARLNLKGRTNAWQPKVNNKDQWLQIDLQHLTKITSIITQGATSMTTAMYVKTFSIHYTDDNST
P. textilis F5	KNSEITASSYKKTWWSSWEPSLARLNLKGRTNAWQPKVNNKDQWLQIDLQHLTKITSIITQGATSMTTSMYVKTFSIHYTDDNST
O. microlepidotus F5	KDSEITASSYKKTWWSSWEPFLARLNLKGRTNAWQPKVNNKDQWLQIDLQHLTKITSIITQGATSMTTAMYVKTFSIHYTDDNST
N. scutatus F5	KNSEITASSYKKTWWSSWEPSLARLNLKGRTNAWQPKVNNKDQWLQIDLQHLTKITSIITQGATSMSTAMYVKTFSIHYTDDNST
B. candidus F5	KNSEITASSYKKTWWSSWEPSLARLNLKGRTNAWQPKVNNKDQWLQIDLQHLTKITSIVTQGATSMSTPMYVKTFSIHYSDDNST
N. helvetica F5	KNSEITASSYKKTWWSSWEPSLARLNLKGRTNAWQPKVNNKDQWLQIDLKHLTKITSIITQGATSMSTEMYVKTFSIHYSDENST

	1871 … 1931
P. textilis vF5 (short)	WKPYLDVRTSMEKVFTGNINSDGHVKHFFKPPILSRFIRIIPKTWNQYIALRIELFGCEVF
O. microlepidotus vF5 (short)	WKPYLDVRTSMEKVFTGNINSDGHVKHFFKPPILSRFIRIIPKTWNQYIALRIELFGCEVF
P. textilis F5 (short)	WKPYLDVRTSMEKVFTGNINSDGHVKHFFKPPILSRFIRIIPKTWNQYIALRIELFGCEVF
O. microlepidotus F5 (short)	WKPYLDVRTSMEKVFTGNINTDGHVKHFFKPPILSRFIRIIPKTWNQYIALRIELFGCEVF
P. textilis F5	WKPYLDVRTSMEKVFTGNINSDGHVKHFFKPPILSRFIRIIPKTWNQYIALRIELFGCEVF
O. microlepidotus F5	WKPYLDVRTSMEKVFTGNINTDGHVKHFFKPPILSRFIRIIPKTWNQYIALRIELFGCEVF
N. scutatus F5	WKPYLDVCTSMEKVFTGNINTDGHVKHFFKPPILSRFIRIIPKTWNQYIALRIELFGCEVF
B. candidus F5	WKPYLDVCTSMEKVFTGNMNTDGHVKHFFIPPILSRFIRIIPKTWNQYIALRIELFGCDVF
N. helvetica F5	WKPYLDVCTSTEKVFTGNVNTDGHVKHFFKPPILSRFIRIIPKTWNRYIALRIELFGCDVF

Fig S8. The alternatively spliced venom factor V protein lacks a large portion of its B-domain region. Amino acid sequence alignment of hypothetical protein translations of *F5* genes**.** The canonical coagulation factor V isoforms are denoted as *vF5* or *F5*, whereas the shorter alternatively spliced isoforms are denoted as *vF5* short or *F5* short. Residues shown in grey correspond to the signal peptide, and residues shown in bold indicate the B-domain region.

O.microlepidotus vF5	MGRYSVSPVPKCLLLMFLGWSGLKYYQVNAAQLREYRIAAQLEDWDY**N**PQPEELSRLSESELTFKKIVYREYELDFKQEKPRDEL
O. microlepidotus vF5 (short)	MGRYSVSPVPKCLLLMFLGWSGLKYYQVNAAQLREYRIAAQLEDWDY**N**PQPEELSRLSESELTFKKIVYREYELDFKQEKPRDEL
O. microlepidotus F5	MGRYSVSPVPKCLLLMFLGWSGLKYYQVNAAQLREYRIAAQLEDWDY**S**PQPEELSRLSESELTFKKIVYREYELDFKQEKPRDEL
transcript/12353	MGRYSVSPVPKCLLLMFLGWSGLKYYQVNAAQLREYRIAAQLEDWDY**N**PQPEELSRLSESELTFKKIVYREYELDFKQEKPRDEL
O. microlepidotus F5 (short)	MGRYSVSPVPKCLLLMFLGWSGLKYYQVNAAQLREYRIAAQLEDWDY**S**PQPEELSRLSESELTFKKIVYREYELDFKQEKPRDEL
transcript/61854	MGRYSVSPVPKCLLLMFLGWSGLKYYQVNAAQLREYRIAAQLEDWDY**N**PQPEELSRLSESELTFKKIVYREYELDFKQEKPRDEL

O.microlepidotus vF5	SGLLGPTLRGEVGDILIIYFKNFATQPVSIHPQSAVYNKWSEGSSYSDGTSDVERLDDAVPPGQSFKYVWNITAEIGPKKADPPC
O. microlepidotus vF5 (short)	SGLLGPTLRGEVGDILIIYFKNFATQPVSIHPQSAVYNKWSEGSSYSDGTSDVERLDDAVPPGQSFKYVWNITAEIGPKKADPPC
O. microlepidotus F5	SGLLGPTLRGEVGDILIIYFKNFATQPVSIHPQSAVYNKWSEGSSYSDGTSDVERLDDAVPPGQSFKYVWNITAEIGPKKADPPC
transcript/12353	SGLLGPTLRGEVGDILIIYFKNFATQPVSIHPQSAVYNKWSEGSSYSDGTSDVERLDDAVPPGQSFKYVWNITAEIGPKKADPPC
O. microlepidotus F5 (short)	SGLLGPTLRGEVGDILIIYFKNFATQPVSIHPQSAVYNKWSEGSSYSDGTSDVERLDDAVPPGQSFKYVWNITAEIGPKKADPPC
transcript/61854	SGLLGPTLRGEVGDILIIYFKNFATQPVSIHPQSAVYNKWSEGSSYSDGTSDVERLDDAVPPGQSFKYVWNITAEIGPKKADPPC

O.microlepidotus vF5	LTY**A**YYSHVNMVRDFNSGLIGALLICKEGSLNANGAQKFFNREYVLMFSVFDESKNWYRKPSLQYTINGFANGTLPDVQACAYDH
O. microlepidotus vF5 (short)	LTY**A**YYSHVNMVRDFNSGLIGALLICKEGSLNANGAQKFFNREYVLMFSVFDESKNWYRKPSLQYTINGFANGTLPDVQACAYDH
O. microlepidotus F5	LTY**S**YYSHVNMVRDFNSGLIGALLICKEGSLNANGAQKFFNREYVLMFSVFDESKNWYRKPSLQYTINGFANGTLPDVQACAYDH
transcript/12353	LTY**A**YYSHVNMVRDFNSGLIGALLICKEGSLNANGAQKFFNREYVLMFSVFDESKNWYRKPSLQYTINGFANGTLPDVQACAYDH
O. microlepidotus F5 (short)	LTY**S**YYSHVNMVRDFNSGLIGALLICKEGSLNANGAQKFFNREYVLMFSVFDESKNWYRKPSLQYTINGFANGTLPDVQACAYDH
transcript/61854	LTY**A**YYSHVNMVRDFNSGLIGALLICKEGSLNANGAQKFFNREYVLMFSVFDESKNWYRKPSLQYTINGFANGTLPDVQACAYDH

O.microlepidotus vF5	ISWHLIGMSSSPEIFSVHFNGQTLEQNHYKVSTINLVGGASVTANMSVSRTGKWLISSLVAKHLQAGMYGYLNIKDCG**H**P**N**TLTR
O. microlepidotus vF5 (short)	ISWHLIGMSSSPEIFSVHFNGQTLEQNHYKVSTINLVGGASVTANMSVSRTGKWLISSLVAKHLQAGMYGYLNIKDCG**H**P**N**TLTR
O. microlepidotus F5	ISWHLIGMSSSPEIFSVHFNGQTLEQNHYKVSTINLVGGASVTANMSVSRTGKWLISSLVAKHLQAGMYGYLNIKDCG**N**P**D**TLTR
transcript/12353	ISWHLIGMSSSPEIFSVHFNGQTLEQNHYKVSTINLVGGASVTANMSVSRTGKWLISSLVAKHLQAGMYGYLNIKDCG**N**P**D**TLTR
O. microlepidotus F5 (short)	ISWHLIGMSSSPEIFSVHFNGQTLEQNHYKVSTINLVGGASVTANMSVSRTGKWLISSLVAKHLQAGMYGYLNIKDCG**N**P**D**TLTR
transcript/61854	ISWHLIGMSSSPEIFSVHFNGQTLEQNHYKVSTINLVGGASVTANMSVSRTGKWLISSLVAKHLQAGMYGYLNIKDCG**N**P**D**TLTR

O.microlepidotus vF5	KLSFRELRRIMNWEYFIAAEEITWDYAPEIPSSVDRRYKAQYLDNFSNFIGKKYKKAVFRQY**E**DGNFTKPTYAIWPKERGILGPV
O. microlepidotus vF5 (short)	KLSFRELRRIMNWEYFIAAEEITWDYAPEIPSSVDRRYKAQYLDNFSNFIGKKYKKAVFRQY**E**DGNFTKPTYAIWPKERGILGPV
O. microlepidotus F5	KLSFRELRRIMNWEYFIAAEEITWDYAPEIPSSVDRRYKAQYLDNFSNFIGKKYKKAVFRQY**K**DGNFTKPTYAIWPKERGILGPV
transcript/12353	KLSFRELRRIMNWEYFIAAEEITWDYAPEIPSSVDRRYKAQYLDNFSNFIGKKYKKAVFRQY**K**DGNFTKPTYAIWPKERGILGPV
O. microlepidotus F5 (short)	KLSFRELRRIMNWEYFIAAEEITWDYAPEIPSSVDRRYKAQYLDNFSNFIGKKYKKAVFRQY**K**DGNFTKPTYAIWPKERGILGPV
transcript/61854	KLSFRELRRIMNWEYFIAAEEITWDYAPEIPSSVDRRYKAQYLDNFSNFIGKKYKKAVFRQY**K**DGNFTKPTYAIWPKERGILGPV

O.microlepidotus vF5	I**K**AKVRDT**VT**IVFKNLASRPYSIYVHGVSVSKDAEGAIYPSDPKENITHGKAVEPGQVYTYKWTVLDTDEPTVKDSECITKLYHS
O. microlepidotus vF5 (short)	I**K**AKVRDT**VT**IVFKNLASRPYSIYVHGVSVSKDAEGAIYPSDPKENITHGKAVEPGQVYTYKWTVLDTDEPTVKDSECITKLYHS
O. microlepidotus F5	I**R**AKVRDT**IS**IVFKNLASRPYSIYVHGVSVSKDAEGAIYPSDPKENITHGKAVEPGQVYTYKWTVLDTDEPTVKDSECITKLYHS
transcript/12353	I**R**AKVRDT**IS**IVFKNLASRPYSIYVHGVSVSKDAEGAIYPSDPKENITHGKAVEPGQVYTYKWTVLDTDEPTVKDSECITKLYHS
O. microlepidotus F5 (short)	I**R**AKVRDT**IS**IVFKNLASRPYSIYVHGVSVSKDAEGAIYPSDPKENITHGKAVEPGQVYTYKWTVLDTDEPTVKDSECITKLYHS
transcript/61854	I**R**AKVRDT**IS**IVFKNLASRPYSIYVHGVSVSKDAEGAIYPSDPKENITHGKAVEPGQVYTYKWTVLDTDEPTVKDSECITKLYHS

O.microlepidotus vF5	AVDMTRDIASGLIGPLLVCK**L**KALSVKGVQNKADVEQHAVFAVFDENKSWYLEDNIKKYCSNPSSVKKDDPKFYKSNVMYTLNGY
O. microlepidotus vF5 (short)	AVDMTRDIASGLIGPLLVCK**L**KALSVKGVQNKADVEQHAVFAVFDENKSWYLEDNIKKYCSNPSSVKKDDPKFYKSNVMYTLNGY
O. microlepidotus F5	AVDMTRDIASGLIGPLLVCK**R**KALSVKGVQNKADVEQHAVFAVFDENKSWYLEDNIKKYCSNPSSVKKDDPKFYKSNVMYTLNGY
transcript/12353	AVDMTRDIASGLIGPLLVCK**R**KALSVKGVQNKADVEQHAVFAVFDENKSWYLEDNIKKYCSNPSSVKKDDPKFYKSNVMYTLNGY
O. microlepidotus F5 (short)	AVDMTRDIASGLIGPLLVCK**R**KALSVKGVQNKADVEQHAVFAVFDENKSWYLEDNIKKYCSNPSSVKKDDPKFYKSNVMYTLNGY
transcript/61854	AVDMTRDIASGLIGPLLVCK**R**KALSVKGVQNKADVEQHAVFAVFDENKSWYLEDNIKKYCSNPSSVKKDDPKFYKSNVMYTLNGY

O.microlepidotus vF5	ASDRTEVLGFHQSEVV**Q**WHLTSVGTVDEIVPVHLSGHTFLSKGKHQDILNLFPMSGESATVTMDNLGTWLLSSWGSCEMSNGMRL
O. microlepidotus vF5 (short)	ASDRTEVLGFHQSEVV**Q**WHLTSVGTVDEIVPVHLSGHTFLSKGKHQDILNLFPMSGESATVTMDNLGTWLLSSWGSCEMSNGMRL
O. microlepidotus F5	ASDRTEVLGFHQSEVV**E**WHLTSVGTVDEIVPVHLSGHTFLSKGKHQDILNLFPMSGESATVTMDNLGTWLLSSWGSCEMSNGMRL
transcript/12353	ASDRTEVLGFHQSEVV**E**WHLTSVGTVDEIVPVHLSGHTFLSKGKHQDILNLFPMSGESATVTMDNLGTWLLSSWGSCEMSNGMRL
O. microlepidotus F5 (short)	ASDRTEVLGFHQSEVV**E**WHLTSVGTVDEIVPVHLSGHTFLSKGKHQDILNLFPMSGESATVTMDNLGTWLLSSWGSCEMSNGMRL
transcript/61854	ASDRTEVLGFHQSEVV**E**WHLTSVGTVDEIVPVHLSGHTFLSKGKHQDILNLFPMSGESATVTMDNLGTWLLSSWGSCEMSNGMRL

O.microlepidotus vF5	RFLDANYDDEDEGNEEEEEDDGDIFADIFSPPEVVKKKE**EV**PVNFV**P**DPESD**AL**AKELGLLDDEDN**P**E**Q**S**RS**EQTEDDEEQLMIA
O. microlepidotus vF5 (short)	RFLDANYDDEDEGNEEEEEDDGDIFADIFSPPEVVKKKE**EV**PVNFV**P**DPESD**AL**AKELGLLDDEDN**P**E**Q**S**RS**EQTEDDEEQLMIA
O. microlepidotus F5	RFLDANYDDEDEGNEEEEEDDGDIFADIFSPPEVVKKKE**KD**PVNFV**S**DPESD**KI**AKELGLLDDEDN**Q**E**E**S**HN**EQTEDDEEQLMIA
transcript/12353	RFLDANYDDEDEGNEEEEEDDGDIFADIFSPPEVVKKKE**KD**PVNFV**S**DPESD**KI**AKELGLLDDEDN**Q**E**E**S**HN**EQTEDDEEQLMIA
O. microlepidotus F5 (short)	RFLDANYDDEDEGNEEEEEDDGDIFADIFSPPEVVKKKE**KD**PVNFV**S**DPESD**KI**AKELGLLDDEDN**Q**E**E**S**HN**EQTEDDEEQLMIA
transcript/61854	RFLDANYDDEDEGNEEEEEDDGDIFADIFSPPEVVKKKE**KD**PVNFV**S**DPESD**KI**AKELGLLDDEDN**Q**E**E**S**HN**EQTEDDEEQLMIA

O.microlepidotus vF5	**S**MLGLRSFKGSVAEEEL**KH**TALALEEDAHASGELSLNTHLVLINSTDAMRGNEELGT**Q**NNSYVNASETIYANYTSSTGEILISNI
O. microlepidotus vF5 (short)	**S**MLGLRSFKGSVAEEEL**KH**TALALEEDAHAS--
O. microlepidotus F5	**T**MLGLRSFKGSVAEEEL**NL**TALALEEDAHASGELSLNTHLVLINSTDAMRGNEELGT**R**NNSYVNASETIYANYTSSTGEILISNI
transcript/12353	**T**MLGLRSFKGSVAEEEL**NL**TALALEEDAHASGELSLNTHLVLINSTDAMRGNEELGT**R**NNSYVNASETIYANYTSSTGEILISNI
O. microlepidotus F5 (short)	**T**MLGLRSFKGSVAEEEL**NL**TALALEEDAHAS--
transcript/61854	**T**MLGLRSFKGSVAEEEL**NL**TALALEEDAHAS--

O.microlepidotus vF5	TAMIGILL**S**KKSQNEENITQISDSYKQMLAAEYLKAGRGLQS**K**DIEDLMSPEIIPSLKEQEENTFEKDIWDKKGERQILTEATLH
O. microlepidotus vF5 (short)	---
O. microlepidotus F5	TAMIGILL**S**KKSQNEENITQISDSYKQMLAAEYLKAGRGLQS**Q**DIEDLMSPEIIPSLKEQEENTFEKDIWDKKGERQILTEATLH
transcript/12353	TAMIGILL**F**KKSQNEENITQISDSYKQMLAAEYLKAGRGLQS**Q**DIEDLMSPEIIPSLKEQEENTFEKDIWDKKGERQILTEATLH
O. microlepidotus F5 (short)	---
transcript/61854	---

O.microlepidotus vF5	ALKEMHALFFYVQQKRNLSAFNITSEDMKFLPSTENDSTIDNSKANYFS**YD**YDYDYDYKEEEATAAANVEFSKVIINTISDDKKT
O. microlepidotus vF5 (short)	---
O. microlepidotus F5	ALKEMHALFFYVQQKRNLSAFNITSEDMKFLPSTENDSTIDNSKANYFS--YDYDYDYKEEEATAAANVEFSKVIINTISDDKKT
transcript/12353	ALKEMHALFFYVQQKRNLSAFNITSEDMKFLPSTENDSTIDNSKANYFS--YDYDYDYKEEEATAAANVEFSKVIINTISDDKKT
O. microlepidotus F5 (short)	---
transcript/61854	---

O.microlepidotus vF5	VENNRSEVNLVSVPSIMPQPENLTSDATLDNFVSTTISKTTK**P**WNSSSHQRQKCLPKNTDMEWKKENYDTLADIPNDVDNKFQNS
O. microlepidotus vF5 (short)	---
O. microlepidotus F5	VENNRSEVNLVSVPSIMPQPENLTSDATLDNFVSTTISKTTK**A**WNSSSHQRQKCLPKNTDMEWKKENYDTLADIPNDVDNKFQNS
transcript/12353	VENNRSEVNLVSVPSIMPQPENLTSDATLDNFVSTTISKTTK**A**WNSSSHQRQKCLPKNTDMEWKKENYDTLADIPNDVDNKFQNS
O. microlepidotus F5 (short)	---
transcript/61854	---

O.microlepidotus vF5	SRNASFYQENAMFPGKRKEKTDNSNGQLELGQPGITNKHDSQKDENCTINISQSFIKIQ*KKKKPKVLTPRTSKHLELPRNLNNS
O. microlepidotus vF5 (short)	---
O. microlepidotus F5	SRNASFYQENAMFPGKRKEKTDNSNGQLELGQPGITNKHDSQKDENCTINISQSFIKIQ**R**KKKKPKVLTPRTSKHLELPRNLNNS
transcript/12353	SRNASFYQENAMFPGKRKEKTDNSNGQLELGQPGITNKHDSQKDENCTINISQSFIKIQ**R**KKKKPKVLTPRTSKHLELPRNLNNS
O. microlepidotus F5 (short)	---
transcript/61854	---

O.microlepidotus vF5	QLSNKPNHTKLLNEAKLTRKQDK**L**IVTIGLPVEDGDYQEYEIDNTDNDKSSSGSFEYELMYYNNPYTTDPRIDSNSARN**S**DDIAG
O. microlepidotus vF5 (short)	--DPRIDSNSARN**S**DDIAG
O. microlepidotus F5	QLSNKPNHTKLLNEAKLTRKQDK**F**IVTIGLPVEDGDYQEYEIDNTDNDKSSSGSFEYELMYYNNPYTTDPRIDSNSARN**P**DDIAG
transcript/12353	QLSNKPNHTKLLNEAKLTRKQDK**L**IVTIGLPVEDGDYQEYEIDNTDNDKSSSGSFEYELMYYNNPYTTDPRIDSNSARN**P**DDIAG
O. microlepidotus F5 (short)	--DPRIDSNSARN**P**DDIAG
transcript/61854	--DPRIDSNSARN**P**DDIAG

O.microlepidotus vF5	RYLRTINR**R**NKRRYYIAAEEVLWDYSPIGKSQVRS**LP**AKTTFKKAIFRSYLDDTFQTPSTGGEYEKHLGILGPIIRAEVDDVIEV
O. microlepidotus vF5 (short)	RYLRTINR**R**NKRRYYIAAEEVLWDYSPIGKSQVRS**LP**AKTTFKKAIFRSYLDDTFQTPSTGGEYEKHLGILGPIIRAEVDDVIEV
O. microlepidotus F5	RYLRTINR**G**NKRRYYIAAEEVLWDYSPIGKSQVRS**RA**AKTTFKKAIFRSYLDDTFQTPSTGGEYEKHLGILGPIIRAEVDDVIEV
transcript/12353	RYLRTINR**G**NKRRYYIAAEEVLWDYSPIGKSQVRS**RA**AKTTFKKAIFRSYLDDTFQTPSTGGEYEKHLGILGPIIRAEVDDVIEV
O. microlepidotus F5 (short)	RYLRTINR**G**NKRRYYIAAEEVLWDYSPIGKSQVRS**RA**AKTTFKKAIFRSYLDDTFQTPSTGGEYEKHLGILGPIIRAEVDDVIEV
transcript/61854	RYLRTINR**G**NKRRYYIAAEEVLWDYSPIGKSQVRS**RA**AKTTFKKAIFRSYLDDTFQTPSTGGEYEKHLGILGPIIRAEVDDVIEV

O.microlepidotus vF5	QFRNLASRPYSLHAHGLLYEKSSEGRSYDD**N**SPELFKKDDAIMPNGTYTYVWQVPPRSGPTDNTEKCKSWAYYSGVNPEKDIHSG
O. microlepidotus vF5 (short)	QFRNLASRPYSLHAHGLLYEKSSEGRSYDD**N**SPELFKKDDAIMPNGTYTYVWQVPPRSGPTDNTEKCKSWAYYSGVNPEKDIHSG
O. microlepidotus F5	QFRNLASRPYSLHAHGLLYEKSSEGRSYDD**K**SPELFKKDDAIMPNGTYTYVWQVPPRSGPTDNTEKCKSWAYYSGVNPEKDIHSG
transcript/12353	QFRNLASRPYSLHAHGLLYEKSSEGRSYDD**K**SPELFKKDDAIMPNGTYTYVWQVPPRSGPTDNTEKCKSWAYYSGVNPEKDIHSG
O. microlepidotus F5 (short)	QFRNLASRPYSLHAHGLLYEKSSEGRSYDD**K**SPELFKKDDAIMPNGTYTYVWQVPPRSGPTDNTEKCKSWAYYSGVNPEKDIHSG
transcript/61854	QFRNLASRPYSLHAHGLLYEKSSEGRSYDD**K**SPELFKKDDAIMPNGTYTYVWQVPPRSGPTDNTEKCKSWAYYSGVNPEKDIHSG

O.microlepidotus vF5	LIGPILICQKGMIDKYNRTIDIREFVLFFMVFDEEKSWYFPKSDKST**CE**EKLIGVQS**H**HTFPAINGIPYQLQGLMMYKDENVHWH
O. microlepidotus vF5 (short)	LIGPILICQKGMIDKYNRTIDIREFVLFFMVFDEEKSWYFPKSDKST**CE**EKLIGVQS**H**HTFPAINGIPYQLQGLMMYKDENVHWH
O. microlepidotus F5	LIGPILICQKGMIDKYNRTIDIREFVLFFMVFDEEKSWYFPKSDKST**RA**EKLIGVQS**R**HTFPAINGIPYQLQGLMMYKDENVHWH
transcript/12353	LIGPILICQKGMIDKYNRTIDIREFVLFFMVFDEEKSWYFPKSDKST**RA**EKLIGVQS**R**HTFPAINGIPYQLQGLMMYKDENVHWH
O. microlepidotus F5 (short)	LIGPILICQKGMIDKYNRTIDIREFVLFFMVFDEEKSWYFPKSDKST**RA**EKLIGVQS**R**HTFPAINGIPYQLQGLMMYKDENVHWH
transcript/61854	LIGPILICQKGMIDKYNRTIDIREFVLFFMVFDEEKSWYFPKSDKST**RA**EKLIGVQS**R**HTFPAINGIPYQLQGLMMYKDENVHWH

O.microlepidotus vF5	LLNMGGPKDIHVVNFHGQTFTEEGREDNQLGVLPLLPGTF**A**SIKMKPSKIGTWLLETEVGENQERGMQALFTVIDKDCKLPMGLA
O. microlepidotus vF5 (short)	LLNMGGPKDIHVVNFHGQTFTEEGREDNQLGVLPLLPGTF**A**SIKMKPSKIGTWLLETEVGENQERGMQALFTVIDKDCKLPMGLA
O. microlepidotus F5	LLNMGGPKDIHVVNFHGQTFTEEGREDNQLGVLPLLPGTF**T**SIKMKPSKIGTWLLETEVGENQERGMQALFTVIDKDCKLPMGLA
transcript/12353	LLNMGGPKDIHVVNFHGQTFTEEGREDNQLGVLPLLPGTF**T**SIKMKPSKIGTWLLETEVGENQERGMQALFTVIDKDCKLPMGLA
O. microlepidotus F5 (short)	LLNMGGPKDIHVVNFHGQTFTEEGREDNQLGVLPLLPGTF**T**SIKMKPSKIGTWLLETEVGENQERGMQALFTVIDKDCKLPMGLA
transcript/61854	LLNMGGPKDIHVVNFHGQTFTEEGREDNQLGVLPLLPGTF**T**SIKMKPSKIGTWLLETEVGENQERGMQALFTVIDKDCKLPMGLA

O.microlepidotus vF5	SGIIQDSQISASGHV**E**YWEPKLARLNN**T**G**MF**NAWSIIKKEHE**H**PWIQIDLQRQVVITGIQTQG**TV**QLLKH**S**YTVEYFVTYSKDGQ
O. microlepidotus vF5 (short)	SGIIQDSQISASGHV**E**YWEPKLARLNN**T**G**MF**NAWSIIKKEHE**H**PWIQIDLQRQVVITGIQTQG**TV**QLLKH**S**YTVEYFVTYSKDGQ
O. microlepidotus F5	SGIIQDSQISASGHV**G**YWEPKLARLNN**P**G**KY**NAWSIIKKEHE**L**PWIQIDLQRQVVITGIQTQG**AM**QLLKH**L**YTVEYFVTYSKDGQ
transcript/12353	SGIIQDSQISASGHV**G**YWEPKLARLNN**P**G**KY**NAWSIIKKEHE**H**PWIQIDLQRQVVITGIQTQG**AM**QLLKH**L**YTVEYFVTYSKDGQ
O. microlepidotus F5 (short)	SGIIQDSQISASGHV**G**YWEPKLARLNN**P**G**KY**NAWSIIKKEHE**L**PWIQIDLQRQVVITGIQTQG**AM**QLLKH**L**YTVEYFVTYSKDGQ
transcript/61854	SGIIQDSQISASGHV**G**YWEPKLARLNN**P**G**KY**NAWSIIKKEHE**H**PWIQIDLQRQVVITGIQTQG**AM**QLLKH**L**YTVEYFVTYSKDGQ

O.microlepidotus vF5	NWITFKGRHSETQMHFEGNSDGTTVKENH**I**DPPIIARYIRLHPTKF**Y**N**T**PTFRIELLGCEVEGCSVPLGMESGAIKNSEITASSY
O. microlepidotus vF5 (short)	NWITFKGRHSETQMHFEGNSDGTTVKENH**I**DPPIIARYIRLHPTKF**Y**N**T**PTFRIELLGCEVEGCSVPLGMESGAIKNSEITASSY
O. microlepidotus F5	NWITFKGRHSETQMHFEGNSDGTTVKENH**T**DPPIIARYIRLHPTKF**H**N**R**PTFRIELLGCEVEGCSVPLGMESGAIKDSEITASSY
transcript/12353	NWITFKGRHSETQMHFEGNSDGTTVKENH**I**DPPIIARYIRLHPTKF**H**N**R**PTFRIELLGCEVEGCSVPLGMESGAIKDSEITASSY
O. microlepidotus F5 (short)	NWITFKGRHSETQMHFEGNSDGTTVKENH**T**DPPIIARYIRLHPTKF**H**N**R**PTFRIELLGCEVEGCSVPLGMESGAIKDSEITASSY
transcript/61854	NWITFKGRHSETQMHFEGNSDGTTVKENH**I**DPPIIARYIRLHPTKF**H**N**R**PTFRIELLGCEVEGCSVPLGMESGAIKDSEITASSY

O.microlepidotus vF5	KKTWWSSWEPFLARLNL**E**G**G**TNAWQP**E**VNNKDQWLQIDLQHLTKITSIITQGATSMTTAMYVKTFSIHYTDDNSTWKPYLDVRTS
O. microlepidotus vF5 (short)	KKTWWSSWEPFLARLNL**E**G**G**TNAWQP**E**VNNKDQWLQIDLQHLTKITSIITQGATSMTTAMYVKTFSIHYTDDNSTWKPYLDVRTS
O. microlepidotus F5	KKTWWSSWEPFLARLNL**K**G**R**TNAWQP**K**VNNKDQWLQIDLQHLTKITSIITQGATSMTTAMYVKTFSIHYTDDNSTWKPYLDVRTS
transcript/12353	KKTWWSSWEPFLARLNL**K**G**R**TNAWQP**K**VNNKDQWLQIDLQHLTKITSIITQGATSMTTAMYVKTFSIHYTDDNSTWKPYLDVRTS
O. microlepidotus F5 (short)	KKTWWSSWEPFLARLNL**K**G**R**TNAWQP**K**VNNKDQWLQIDLQHLTKITSIITQGATSMTTAMYVKTFSIHYTDDNSTWKPYLDVRTS
transcript/61854	KKTWWSSWEPFLARLNL**K**G**R**TNAWQP**K**VNNKDQWLQIDLQHLTKITSIITQGATSMTTAMYVKTFSIHYTDDNSTWKPYLDVRTS

O.microlepidotus vF5	MEKVFTGNIN**S**DGHVKHFFKPPILSRFIRIIPKTWNQYIALRIELFGCEVF
O. microlepidotus vF5 (short)	MEKVFTGNIN**S**DGHVKHFFKPPILSRFIRIIPKTWNQYIALRIELFGCEVF
O. microlepidotus F5	MEKVFTGNIN**T**DGHVKHFFKPPILSRFIRIIPKTWNQYIALRIELFGCEVF
transcript/12353	MEKVFTGNIN**T**DGHVKHFFKPPILSRFIRIIPKTWNQYIALRIELFGCEVF
O. microlepidotus F5 (short)	MEKVFTGNIN**T**DGHVKHFFKPPILSRFIRIIPKTWNQYIALRIELFGCEVF
transcript/61854	MEKVFTGNIN**T**DGHVKHFFKPPILSRFIRIIPKTWNQYIALRIELFGCEVF

Fig S9. Full-length *F5* transcripts are present in inland taipan (*Oxyuranus microlepidotus*) venom gland tissue. Amino acid alignment of the hypothetical protein isoform translations encoded by the *F5* and *vF5* paralogs of *O. microlepidotus*, together with representative full-length translated Iso-seq reads of both isoforms from venom gland tissue corresponding to each *F5* paralog. The alignment provides qualitative evidence that both *F5* alternative splice variants are expressed in venom gland tissue. A single-base-pair indel in transcript/12353 that was absent in all other transcripts and therefore likely represents a technical artifact, was manually corrected to restore the reading frame. Amino acid residues that differ between the *F5* and vF5 genes are shown in bold.

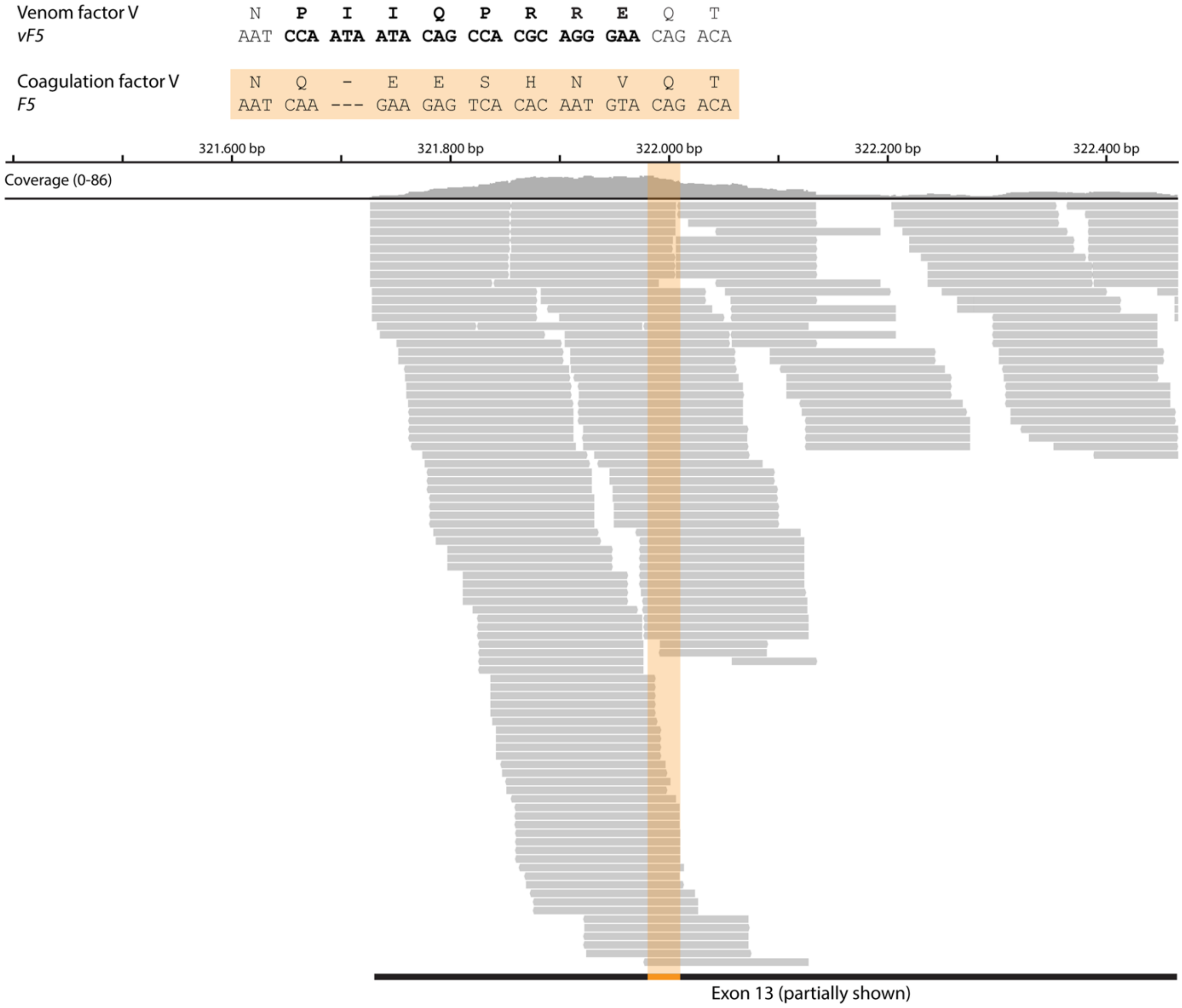

Fig S10. The hemostatic *F5* gene is expressed in Eastern brown snake (*Pseudonaja textilis*) venom gland tissue. One distinguishing feature between the *F5* and *vF5* paralogs in *P. textilis* is a unique structural variant in exon 13 of the *vF5* gene. The unique protein sequence and underlying codons are shown in bold for the *vF5* gene, with the corresponding protein sequence and codons in the *F5* gene highlighted in orange. We identified multiple short-read RNA-seq reads from *P. textilis* venom gland tissue that map to/cover this *F5*-specific region (highlighted in orange), providing additional evidence that the hemostatic *F5* gene is also transcribed in the venom gland of *P. textilis*.

Pseudonaja textilis vF5	MGRYSVSPVPKCLLLMFLGWSGLKYYQVNAAQLREYHIAAQLEDWDYNPQPEELSR**LSESDLTFKK**IVYR**EYELDFK**QEKPRDA
Pseudonaja textilis F5 (short)	MGRYSVSPVPKCLLLMFLGWSGLKYYQVNAAQLREYRIAAQLEDWDYNPQPEELSR**LSESDLTFKK**IVYR**EYELDFK**QEKPRDA
Pseudonaja textilis F5	MGRYSVSPVPKCLLLMFLGWSGLKYYQVNAAQLREYRIAAQLEDWDYNPQPEELSR**LSESDLTFKK**IVYR**EYELDFK**QEKPRDA
Pseudonaja textilis vF5	LSGLLGPTLRGEVGDSLIIYFKNFATQPVSIHPQSAVYNK**WSEGSSYSDGTSDVERLDDAVPPGQSFK**YVWNITAEIGPKKADP
Pseudonaja textilis F5 (short)	LSGLLGPTLRGEVGDILIIYFKNFATQPVSIHPQSAVYNK**WSEGSSYSDGTSDVERLDDAVPPGQSFK**YVWNITAEIGPKKADP
Pseudonaja textilis F5	LSGLLGPTLRGEVGDILIIYFKNFATQPVSIHPQSAVYNK**WSEGSSYSDGTSDVERLDDAVPPGQSFK**YVWNITAEIGPKKADP
Pseudonaja textilis vF5	PCLTYAYYSHVNMVRDFNSGLIGALLICK**EGSLNANGSQK**FFNREYVLMFSVFDESKNWYRKPSLQYTINGFANGTLPDVQACA
Pseudonaja textilis F5 (short)	PCLTYAYYSHVNMVRDFNSGLIGALLICK**EGSLNANGAQK**FFNREYVLMFSVFDESKNWYRKPSLQYTINGFANGTLPDVQACA
Pseudonaja textilis F5	PCLTYAYYSHVNMVRDFNSGLIGALLICK**EGSLNANGAQK**FFNREYVLMFSVFDESKNWYRKPSLQYTINGFANGTLPDVQACA
Pseudonaja textilis vF5	YDHISWHLIGMSSSPEIFSVHFNGQTLEQNHYKVSTINLVGGASVTADMSVSRTGKWLISSLVAKHLQAGMYGYLNIK**DCGNPD**
Pseudonaja textilis F5 (short)	YDHISWHLIGMSSSPEIFSVHFNGQTLEQNHYKVSTINLVGGASVTANMSVSRTGKWLISSLVAKHLQAGMYGYLNIK**DCGNPD**
Pseudonaja textilis F5	YDHISWHLIGMSSSPEIFSVHFNGQTLEQNHYKVSTINLVGGASVTANMSVSRTGKWLISSLVAKHLQAGMYGYLNIK**DCGNPD**
Pseudonaja textilis vF5	**TLTR**KLSFRELMKIKNWEYFIAAEEITWDYAPEIPSSVDRRYKAQYLDNFSNFIGKKYKKAVFRQYEDGNFTKPTYAIWPKER**G**
Pseudonaja textilis F5 (short)	**TLTR**KLSFRELRRIMNWEYFIAAEEITWDYAPEIPSSVDRRYKAQYLDNFSNFIGKKYKKAVFR**QYKDSNFTK**PTYAIWPKERG
Pseudonaja textilis F5	**TLTR**KLSFRELRRIMNWEYFIAAEEITWDYAPEIPSSVDRRYKAQYLDNFSNFIGKKYKKAVFR**QYKDSNFTK**PTYAIWPKERG
Pseudonaja textilis vF5	**ILGPVIK**AK**VRDTVTIVFK**NLASRPYSIYVHGVSVSKDAEGAIYPSDPKENITHGK**AVEPGQVYTYK**WTVLDTDEPTVK**DSECI**
Pseudonaja textilis F5 (short)	ILGPVIRAK**VRDTISIVFK**NLASRPYSIYVHGVSVSKDAEGAIYPSDPKENITHGK**AVEPGQVYTYK**WTVLDTDEPTVK**DSECI**
Pseudonaja textilis F5	ILGPVIRAK**VRDTISIVFK**NLASRPYSIYVHGVSVSKDAEGAIYPSDPKENITHGK**AVEPGQVYTYK**WTVLDTDEPTVK**DSECI**
Pseudonaja textilis vF5	**TKLYHSAVDMTR**DIASGLIGPLLVCKHKALSVKGVQNKADVEQHAVFAVFDENKSWYLEDNIK**KYCSNPSAVKKDDPKFYK**SNV
Pseudonaja textilis F5 (short)	**TKLYHSAVDMTR**DIASGLIGPLLVCKHKALSVKGVQNKADVEQHAVFAVFDENKSWYLEDNIK**KYCSNPSTVKKDDPKFYK**SNV
Pseudonaja textilis F5	**TKLYHSAVDMTR**DIASGLIGPLLVCKHKALSVKGVQNKADVEQHAVFAVFDENKSWYLEDNIK**KYCSNPSTVKKDDPKFYK**SNV
Pseudonaja textilis vF5	MYTLNGYASDRTEVLRFHQSEVVQWHLTSVGTVDEIVPVHLSGHTFLSKGKHQDILNLFPMSGESATVTMDNLGTWLLSSWGSC
Pseudonaja textilis F5 (short)	MYTLNGYASDRTEVLGFHQSEVVEWHLTSVGTVDEIVPVHLSGHTFLSKGKHQDILNLFPMSGESATVTMDNLGTWLLSSWGSC
Pseudonaja textilis F5	MYTLNGYASDRTEVLGFHQSEVVEWHLTSVGTVDEIVPVHLSGHTFLSKGKHQDILNLFPMSGESATVTMDNLGTWLLSSWGSC
Pseudonaja textilis vF5	EMSNGMRLRFLDANYDDEDEGNEEEEEDDGDIFADIFIPSEVVKKKEEVPVNFVPDPESDALAKELGLIDDEGNPIIQPR**REQT**
Pseudonaja textilis F5 (short)	EMSNGMRLRFLDANYDDEDEGNEEEEEDDGDIFADIFIPPEVVKKKEKDPVNFVSDPESDKIAKELGLLDDEDNQ-EESHNVQT
Pseudonaja textilis F5	EMSNGMRLRFLDANYDDEDEGNEEEEEDDGDIFADIFIPPEVVKKKEKDPVNFVSDPESDKIAKELGLLDDEDNQ-EESHNVQT
Pseudonaja textilis vF5	**EDDEEQLMK**ASMLGLRSFKGSVAEEELKHTALALEEDAHAS---
Pseudonaja textilis F5 (short)	EDDEEQLMIATMLGFRSFKGSVAEEELNLTALALEEDAHAS---
Pseudonaja textilis F5	EDDEEQLMIATMLGFRSFKGSVAEEELNLTALALEEDAHASGELSLNTHLVLINSTDAMRGNEELGTQNNSYVNASETIYANYT
Pseudonaja textilis vF5	--
Pseudonaja textilis F5 (short)	--
Pseudonaja textilis F5	SSTGEILISNITAMIGILLSKRSQNEENITQISDSYKEMLAAEYLKAGRGLQSKDIEDLMSPEIIPSLKEQEENTFEKDIWDKK
Pseudonaja textilis vF5	--
Pseudonaja textilis F5 (short)	--
Pseudonaja textilis F5	GERQILTEATLHALKEMHALFFYVQQKRNLSAFNITSEGMKFLPSTENDSTIDNSKANYFSYDYDYDYKEEEATAAANVEFSKV
Pseudonaja textilis vF5	--
Pseudonaja textilis F5 (short)	--
Pseudonaja textilis F5	IINTISDDKKTVENNRSEVNLVSVPSIMPQPENLTSDATLDNFVSTTISKTTKTWNSSSHQRQKCLPKNTDMEWKKENYDTLAD
Pseudonaja textilis vF5	--
Pseudonaja textilis F5 (short)	--
Pseudonaja textilis F5	IPNDVDNKFQNSSRNASFYQENAMFPGKRKEKTDNSNGQLELGQPGITNKHDSQKDENCTINISQSFIKIQRKKKKPKVLTPRT
Pseudonaja textilis vF5	---DPR
Pseudonaja textilis F5 (short)	---DPR
Pseudonaja textilis F5	SKHLELPRNLNNSQLSNKPNHTKLLNEAKLTRKQDKLIVTIGLPVEDGDYQEYDIDNTDNDRSSSGSFEYELMYYNNPYTTDPR
Pseudonaja textilis vF5	**IDSNSARNPDDIAGR**YLRTINRGNKRRYYIAAEEVLWDYSPIGKSQVRSRAAKTTFKKAIFRSYLDDTFQTPSTGGEYEKHLGI
Pseudonaja textilis F5 (short)	**IDSNSARNPDDIAGR**YLRTINRGNKRRYYIAAEEVLWDYSPIGKSQVRSRAAKTTFKKAIFRSYLDDTFQTPSTGGEYEKHLGI
Pseudonaja textilis F5	**IDSNSARNPDDIAGR**YLRTINRGNKRRYYIAAEEVLWDYSPIGKSQVRSRAAKTTFKKAIFRSYLDDTFQTPSTGGEYEKHLGI
Pseudonaja textilis vF5	LGPIIRAEVDDVIEIQFRNLASR**PYSLHAHGLLYEK**SSEGR**SYDDKSPELFKK**DDAIMPNGTYTYVWQVPPR**SGPTDNTEK**CKS
Pseudonaja textilis F5 (short)	LGPIIRAEVDDVIEVQFRNLASR**PYSLHAHGLLYEK**SSEGR**SYDDKSPELFKK**DDAIMPNGTYTYVWQVPPR**SGPTDNTEK**CKS
Pseudonaja textilis F5	LGPIIRAEVDDVIEVQFRNLASR**PYSLHAHGLLYEK**SSEGR**SYDDKSPELFKK**DDAIMPNGTYTYVWQVPPR**SGPTDNTEK**CKS
Pseudonaja textilis vF5	WAYYSGVNPEKDIHSGLIGPILICQK**GMIDKYNR**TIDIREFVLFFMVFDEEKSWYFPKSDKSTCEEKLIGVQSLHTFPAINGIP
Pseudonaja textilis F5 (short)	WAYYSGVNPEKDIHSGLIGPILICQK**GMIDKYNR**TIDIREFVLFFMVFDEEKSWYFPKSDKSTRAEKLIGVQSRHTFPAINGIP
Pseudonaja textilis F5	WAYYSGVNPEKDIHSGLIGPILICQK**GMIDKYNR**TIDIREFVLFFMVFDEEKSWYFPKSDKSTRAEKLIGVQSRHTFPAINGIP
Pseudonaja textilis vF5	YQLQGLTMYKDENVHWHLLNMGGPK**DIHVVNFHGQTFTEEGR**EDNQLGVLPLLPGTFASIKMKPSKIGTWLLETEVGENQERGM
Pseudonaja textilis F5 (short)	YQLQGLTMYKDENVHWHLLNMGGPK**DIHVVNFHGQTFTEEGR**EDNQLGVLPLLPGTFASIKMKPSKIGTWLLETEVGENQERGM
Pseudonaja textilis F5	YQLQGLTMYKDENVHWHLLNMGGPK**DIHVVNFHGQTFTEEGR**EDNQLGVLPLLPGTFASIKMKPSKIGTWLLETEVGENQERGM

Pseudonaja textilis vF5	QALFTVIDKDCKLPMGLASGIIQDSQISASGHVGYWEPKLARLNNTAIFNAWSIIKKEHEHPWIQIDLQRQVVITGIQTQGTVQ
Pseudonaja textilis F5 (short)	QALFTVIDKDCKLPMGLASGIIQDSQISASGHVGYWEPKLARLNNTGKYNAWSIIKKEHEHPWIQIDLQRQVVITGIQTQGAMQ
Pseudonaja textilis F5	QALFTVIDKDCKLPMGLASGIIQDSQISASGHVGYWEPKLARLNNTGKYNAWSIIKKEHEHPWIQIDLQRQVVITGIQTQGAMQ
Pseudonaja textilis vF5	LLQHSYTVEYFVTYSEDGQNWITFK**GRHSETQMHFEGNSDGTTVKENHIDPPIIAR**YIRLHPTK**FYNRPTFR**IELLGCEVEGCS
Pseudonaja textilis F5 (short)	LLKHLYTVEYFFTYSKDGQNWITFK**GRHSETQMHFEGNSDGTTVKENHIDPPIIAR**YIRLHPTKFHNRPTFRIELLGCEVEGCS
Pseudonaja textilis F5	LLKHLYTVEYFFTYSKDGQNWITFK**GRHSETQMHFEGNSDGTTVKENHIDPPIIAR**YIRLHPTKFHNRPTFRIELLGCEVEGCS
Pseudonaja textilis vF5	VPLGMESGAIK**NSEITASSYKK**TWWSSWEPFLARLNLEGGTNAWQPEVNNKDQWLQIDLQHLTKITSIITQGATSMTTSMYVKT
Pseudonaja textilis F5 (short)	VPLGMESGAIK**NSEITASSYKK**TWWSSWEPSLARLNLKGRTNAWQPKVNNKDQWLQIDLQHLTKITSIITQGATSMTTSMYVKT
Pseudonaja textilis F5	VPLGMESGAIK**NSEITASSYKK**TWWSSWEPSLARLNLKGRTNAWQPKVNNKDQWLQIDLQHLTKITSIITQGATSMTTSMYVKT
Pseudonaja textilis vF5	FSIHYTDDNSTWKPYLDVRTSMEK**VFTGNINSDGHVKHFFKPPILSR**FIRIIPKTWNQYIALRIELFGCEVF
Pseudonaja textilis F5 (short)	FSIHYTDDNSTWKPYLDVRTSMEK**VFTGNINSDGHVKHFFKPPILSR**FIRIIPKTWNQYIALRIELFGCEVF
Pseudonaja textilis F5	FSIHYTDDNSTWKPYLDVRTSMEK**VFTGNINSDGHVKHFFKPPILSR**FIRIIPKTWNQYIALRIELFGCEVF

Fig. S11. Coagulation factor V protein is detected in Eastern brown snake (*Pseudonaja textilis*) venom. Amino acid sequence alignment of hypothetical F5 protein translations with tryptic peptides shown in bold. Red residues indicate peptides unique to the venom protein (vF5), purple residues indicate peptides unique to the hemostatic liver isoforms (F5 or F5 short), and black residues indicate peptides shared by both paralogs. Residues shown in grey correspond to the signal peptide. Tryptic peptides corresponding to the hemostatic form were detected in venoms from all individuals (N = 7).

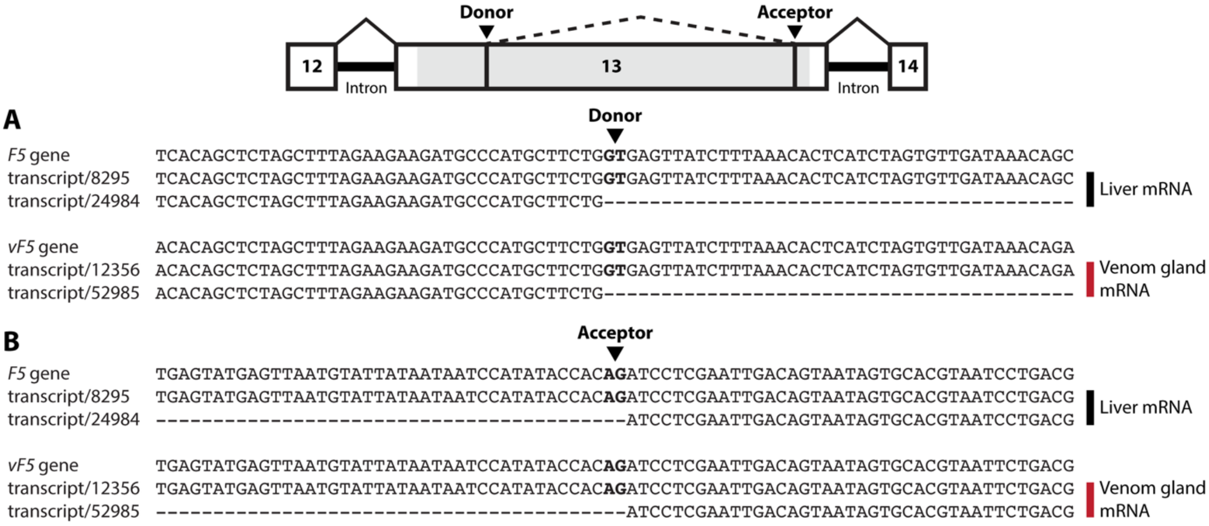

Fig. S12. *F5* gene paralogs are alternatively spliced within exon 13. DNA and mRNA sequence comparison at the donor (A) and acceptor (B) splice sites within exon 13 of both *F5* paralogs. The alignments include the *F5* and *vF5* genes as references with each of their corresponding full-length RNA transcripts expressed in liver or venom gland tissue of the inland taipan (*Oxyuranus microlepidotus*). The longer RNA isoform encodes the entire exon 13 (transcripts 8295 and 12356), whereas the shorter isoform (transcripts 24984 and 52985) retains only two short coding sequences at the 5′ and 3′ ends of exon 13. These segments are flanked by two canonical splice sites (donor GT and acceptor AG), indicating that the shorter isoform is generated through alternative splicing within exon 13.

	1 — 76
P. textilis Acidic PLA2 1	MHPAHLLVLLGVYVSLLGAARIPPLPLSLDDFSNLITCANRGSRSWLDYAHYGCFCGSGGSGT
P. textilis Acidic PLA2 2	MHPAHLLVLLGVCVSLLGAARIPPLPLSLVQFRILIKCANHNSRNVLDYADYGCYCGKGGSGT
O. microlepidotus Acidic PLA2 1 OS1	MHPAHLLVLLAVCVSLLGAARIPPLPLSLLDFANLIECANHGTRSALAYVHYGCYCGKGGRGT
N. scutatus Acidic PLA2 HTe	MYPAHLQVLLAVCVSLLGASSIPARPLNLYQFGNMIQCANHGRRPTRHYMDYGCYCGKGGSGT
P. textilis Acidic PLA2 textilotoxin D subunit	MHPAHLLVLLGVCVSRLGAASIPRPSLNIMLFGNMIQCTIPCEQSWLGYLDYGCYCGSGSSGI
O. microlepidotus Acidic PLA2 taipoxin γ subunit	MHPAHLLVLLAVCVSLLGSSEIPQPSLDFEQFSNMIQCTIPCGESCLAYMDYGCYCGPGGSGT
B. candidus Acidic PLA2 Bc-PL	MNPAHLLVLSALCVSLLGAANIPPQPLNLLQFNEMIECTIPGSFPLLDYMDYGCYCGTGGRGT
B. candidus Acidic PLA2 1	MNPAHFLVLAAVCVSLLGAANIPPQPLSFYRYTQMIQCTIRGSLTYLDYMDYGCYCGTGSRGT
B. candidus Acidic β bungarotoxin A3	MNPAHLLVLAAVCVSLLGAANIPPHPLNLINFMEMIRYTIPCEKTWGEYTNYGCYCGAGGSGR
B. candidus Basic β bungarotoxin A2	MNPAHLLVLSAVCVSLLGAANIPPHPFNLINFMKMIRYTIPCEKTWGEYVDYGCYCGVGGSGR
B. candidus Basic β bungarotoxin A1	MNPAHLLVLSAVCVSLLGAANIPPHPLNLINFMEMIRYTIPCEKTWGEYADYGCYCGAGGSGR
N. scutatus Basic PLA2 notechis 11'2	MYPAHLLVLLAVCVSLLGAASIPPQPLNLYQFGNMIQCANHGSRATWHYMDYGCYCGAGGSGT
N. scutatus Basic PLA2 notexin	MYPAHLLVLLAVCVSLLGAASIPPQPLNLVQFSYLIQCANHGSRATWHYMDYGCYCGAGGSGT
N. scutatus PLA2 1	MYPAHLLVLLAVCVSLLGATSIPPQPLNLYQFKNMIPCANHGRRPTWHYMDYGCYCSSGGSGT
N. scutatus Acidic PLA2	MYPAHLLVLLAVCVSLLGAASIPPLPLNVAQFDNMIECANYGSRPSWHYMEYGCYCGKEGSGT
P. textilis Basic PLA2 textilotoxin A subunit	MHPAHLLVLLGVCVSLLGASDIPPLPLNLVQFSYLIRCANKYKRPGWHYANYGCYCGSGGRGT
P. textilis Basic PLA2 textilotoxin B subunit	MHPAHLLVLLAVYVSLLGAARIPPLPLDLVEFGFMIRCANRNSQPAWQYMDYGCYCGKRGSGT
O. microlepidotus Neutral PLA2 taipoxin β subunit	MHPAHLLVLLAVCVSLLGASDIPPLPLNLLQFGFMIECAIRNRQPALDFMNYGCYCGTVGRGT
P. textilis Acidic PLA2 textilotoxin C subunit	MHPAHLLVLLGVYVSLLGAARIPPLPLNLIQFSNMIKCTIPGSQPLLDYANYGCYCGPGNNGT
O. microlepidotus Basic PLA2 taipoxin α subunit 1	MHPAHLLVLLAVCVSLLGASDIPPLPLNLLQFGFMIRCANRRSRPVWHYMDYGCYCGKGGRGT
O. microlepidotus Basic PLA2 taipoxin α subunit 2	MHPAHLLVLLAVCVSLLGASDIPPLPLNLAQFGFMIKCANGGSRPVSHYMDYGCYCGKGGSGT
O. microlepidotus Basic PLA2 taipoxin α subunit 3	MHPAHLLVLLAVCVSLLGASDIPPLPLNLAQFGFMIKCANGGSRPVSHYMDYGCYCGKGGSGT
O. microlepidotus Basic PLA2 taipoxin α subunit 4	MHPAHLLVLLAVCVSLLGASDIPPLPLNLAQFGFMIKCANGGSRPVSHYMDYGCYCGKGGSGT
O. microlepidotus Basic PLA2 taipoxin α subunit 5	MHPAHLLVLLAVCVSLLGASDIPPLPLNLAQFGFMIKCANGGSRPVSHYMDYGCYCGKGGSGT
O. microlepidotus Basic PLA2 taipoxin α subunit 6	MHPAHLLVLLAVCVSLLGASDIPPLPLNLAQFGFMIKCANGGSRPVSHYMDYGCYCGKGGSGT
P. textilis Group IB PLA2	MNPAHLLVLLAVCVSLLGASDIPPRPLNLIQFGNMIKCTIPGSRPLLDYADYGCYCGPGGSGT
O. microlepidotus Group IB PLA2	MNPAHLLVLLAVCVSLLGASDIPPLPLNLIQFGNMIKCTIPGSRPLLDYADYGCYCGPGGSGT
B. candidus Group IB PLA2	MNPAHLLVLSAVCVSLLGAANVAPQPRNLYQFRNMIKCTIPGSRPLLDYANYGCYCGKGGRGT
N. helvetica Group IB PLA2	MNLAHLLVPAALCVSLVAASALPSLPRNLLQFSNMIKCAIPGSRPVLDYADYGCYCGFGGSGT

	77 — 126
P. textilis Acidic PLA2 1	PVDDLDRCCQVHDNCFGDAEKLPACN**YLFSG**PYWNPYSYKCNEGE-ITCTDDNDECAAFICNC
P. textilis Acidic PLA2 2	PVDELDRCCQAHDYCYDDAEKLPACN**YRFSG**PYWNPYSYKCNEGE-VTCTDDNDECKAFICNC
O. microlepidotus Acidic PLA2 1 OS1	PVDDLDRCCQVHDDCYGEAEKLPACN**YLMSS**PYFNSYSYKCNEGK-VTCTDDNDECKAFICNC
N. scutatus Acidic PLA2 HTe	PVDELDRCCKTHDDCYGEAEKLPACN**YMMSG**PYYNTYSYECNEGE-LTCKDDNDECGAFICNC
P. textilis Acidic PLA2 textilotoxin D subunit	PVDDVDKCCKTHDECYYKAGQIPGCS**VQPNE**VFNVDYSYECNEGQ-LTCNESNNECEMAVCNC
O. microlepidotus Acidic PLA2 taipoxin γ subunit	PIDDLDRCCKTHDECYAEAGKLSACK**SVLSE**PNNDTYSYECNEGQ-LTCNDDNDECKAFICNC
B. candidus Acidic PLA2 Bc-PL	PVDMLDRCCKEHDDCYAQIKENPKCS**SLLNV**PYVKQYSFTCSEGD-LTCSDAAGTCARIVCDC
B. candidus Acidic PLA2 1	PVDELDRCCQTHDNCYAEAEEHPKCS**SLVKS**PYMNLYSYTCSGGT-ITCNADNDECGAFICNC
B. candidus Acidic β bungarotoxin A3	PIDALDRCCYVHDNCYGDAANIRDC-----**N**PKTQSYSYKLTKRT-IICYGAAGTCARVVCDC
B. candidus Basic β bungarotoxin A2	PIDALDRCCYVHDNCYGDAEKKHKC-----**N**PKMQSYSYKLTKRT-IICYGAAGTCARIVCDC
B. candidus Basic β bungarotoxin A1	PIDALDRCCYVHDNCYGDAEKKHKC-----**N**PKTQSYSYKLTKRT-IICYGAAGTCARIVCDC
N. scutatus Basic PLA2 notechis 11'2	PVDELDRCCKAHDDCYTEAGKK-GC-----**S**PKLTLYSWKCIEKT-PTC-NSKTGCERSVCDC
N. scutatus Basic PLA2 notexin	PVDELDRCCKIHDDCYGDAEKK-GC-----**S**PKMSAYDYYCGENG-PYCRNIKKKCLRFVCDC
N. scutatus PLA2 1	PVDELDRCCKTHDDCYTNIGTS-KC-----**N**PKSQIYSWKCGSDG-PTC-DSKTGCERSVCEC
N. scutatus Acidic PLA2	PVDELDRCCKAHDDCYTEAEKR-RC-----**H**PKFSAYSWKCGSDG-PTC-DSETGCKRTVCDC
P. textilis Basic PLA2 textilotoxin A subunit	PVDDVDRCCQAHDKCYEDAEKL-GC-----**Y**PKWTTYYYYCGANG-PYC-KTRTKCQRFVCNC
P. textilis Basic PLA2 textilotoxin B subunit	PVDDVDRCCQTHNECYDEAAKIPGC-----**K**PKWTFYFWQCGSGSQFTCRKSKDVCRNVVCDC
O. microlepidotus Neutral PLA2 taipoxin β subunit	PVDDLDRCCKTRNECYAEAEKH-GC-----**Y**PSLTTYRWQCGRVG-LHC-NSKTQCEVFVCAC
P. textilis Acidic PLA2 textilotoxin C subunit	PVDDVDRCCQAHDECYDEASNH-GC-----**Y**PELTLYDYYCDTGV-PYC-KARTQCQVFVCGC
O. microlepidotus Basic PLA2 taipoxin α subunit 1	PVDDLDRCCQVHDECYGEAERRFGC-----**S**PYWTLYSWKCYGTA-PSC-NTKTRCQRFVCRC
O. microlepidotus Basic PLA2 taipoxin α subunit 2	PVDELDRCCQVHDECYGEAEKRFKC-----**V**PYMTLYSWKCYGTA-PSC-NTKTDCQRFVCNC
O. microlepidotus Basic PLA2 taipoxin α subunit 3	PVDELDRCCQVHDECYGEAEKRFKC-----**V**PYMTLYSWKCYGTA-PSC-NTKTDCQRFVCNC
O. microlepidotus Basic PLA2 taipoxin α subunit 4	PVDELDRCCQVHDECYGEAEKRFKC-----**V**PFVTLYSWKCYGKA-PSC-NTKTDCQRFVCNC
O. microlepidotus Basic PLA2 taipoxin α subunit 5	PVDELDRCCQVHDECYGEAEKRFKC-----**V**PFVTLYSWKCYGKA-PSC-NTKTDCQRFVCNC
O. microlepidotus Basic PLA2 taipoxin α subunit 6	PVDELDRCCQVHDECYGEAEKRFKC-----**V**PYMTLYSWKCYGTA-PSC-NTKTDCQRFVCNC
P. textilis Group IB PLA2	PVDELDRCCQTHDNCYSQAKKHPACK**SLLDS**PYTKIYSYTCSGGN-LTCKDDNDECGAFICNC
O. microlepidotus Group IB PLA2	PVDELDRCCQTHDNCYSQAKKHPACK**SLLDS**PYTKIYSYTCSGGN-LTCKDDNDECGAFICNC
B. candidus Group IB PLA2	PVDMLDRCCQTHDNCYDQAMKHPACK**SFLDS**PYIKTYSYTCSGGT-VTCKDVKDKCARFVCDC
N. helvetica Group IB PLA2	PVDQLDRCCQTHDNCYSQAKKHPACK**SLLDS**PYTKTYSYTCSGGN-ITCQDNNDECEAFVCNC

	127 155
P. textilis Acidic PLA2 1	DRTAAICFAGATYNDENFMVTIKKKNICQ
P. textilis Acidic PLA2 2	DRTAAICFAGAPYNDENFMITTKKKNICQ
O. microlepidotus Acidic PLA2 1 OS1	DRTAAICFAGATYNDENFMISKKRNDICQ
N. scutatus Acidic PLA2 HTe	DRTAAICFARAPYNDANWDINLIAR--CQ
P. textilis Acidic PLA2 textilotoxin D subunit	DRAAAICFARFPYNKNYWSINTEIH--CR
O. microlepidotus Acidic PLA2 taipoxin γ subunit	DRTAVTCFAGAPYNDLNYNIGMIEH--CK
B. candidus Acidic PLA2 Bc-PL	DRTAALCFAEAPYKRRNFKIDYKTR--CQ
B. candidus Acidic PLA2 1	DRTAALCFAKAPYNEENKEIDISKR--CQ
B. candidus Acidic β bungarotoxin A3	DRTAALCFGDSEYIEGHKNIDTARF--CQ
B. candidus Basic β bungarotoxin A2	DRTAALCFGNSEYIERHKNIDTKRY--CR
B. candidus Basic β bungarotoxin A1	DRTAALCFGDSEYIERHKNIDTKRH--CQ
N. scutatus Basic PLA2 notechis 11'2	DAAAAKCFAKAPYNKKNYNINTKKR--CQ
N. scutatus Basic PLA2 notexin	DVEAAFCFAKAPYNNANWNIDTKKR--CQ
N. scutatus PLA2 1	DAAAAKCFAKAPYNDANWNIDTEKH--CQ
N. scutatus Acidic PLA2	DATAAKCFAKAPFNQANWNIDTETH--CQ
P. textilis Basic PLA2 textilotoxin A subunit	DVVAADCFASYPYNRRYWFYSNKKR--CR
P. textilis Basic PLA2 textilotoxin B subunit	DFKAALCLTRARYNSANYNIDIKTH--CR
O. microlepidotus Neutral PLA2 taipoxin β subunit	DLAAAKCLAQEDYNPAHFNINTKAR--CR
P. textilis Acidic PLA2 textilotoxin C subunit	DLAVAKCLAGATYNDENKNINTGER--CQ
O. microlepidotus Basic PLA2 taipoxin α subunit 1	DAKAAECFARSPYQSSNWNINTKAR--CR
O. microlepidotus Basic PLA2 taipoxin α subunit 2	DAKAAECFARSPYQNKNWNINTKAR--CK
O. microlepidotus Basic PLA2 taipoxin α subunit 3	DAKAAECFARSPYQNKNWNINTKAR--CK
O. microlepidotus Basic PLA2 taipoxin α subunit 4	DAKAAECFARSPYQNKNWNINTKAR--CK
O. microlepidotus Basic PLA2 taipoxin α subunit 5	DAKAAECFARSPYQNKNWNINTKAR--CK
O. microlepidotus Basic PLA2 taipoxin α subunit 6	DAKAAECFARSPYQNKNWNINTKAR--CK
P. textilis Group IB PLA2	DRTAAICFAGAPYNKENYNIDTTKH--CK
O. microlepidotus Group IB PLA2	DRTAAICFAGAPYNKENYNIDTTKH--CK
B. candidus Group IB PLA2	DRAAAMCFARAPYNKKYKKLDKSKY--CK
N. helvetica Group IB PLA2	DRSAAICFAGAPYNEEYKKLDTGKY--CK

Fig. S13. The pancreatic loop is intact in group IB but not in group IA phospholipase A_2 venom proteins. Amino acid sequence alignment of hypothetical protein translations of group I phospholipase A_2 (PLA_2) genes. Residues shown in grey encode the signal peptide and propeptide regions, and residues in bold indicate the pancreatic loop in group IB PLA_2, which is absent in group IA PLA_2 proteins because of a 5-amino-acid deletion. The procoagulant proteins are "*P. textilis* Acidic PLA2 1", "*P. textilis* Acidic PLA2 2" and putatively "*O. microlepidotus* Acidic PLA2 1 OS1" based on its orthologous relationship to "*P. textilis* Acidic PLA2 1".

	1 85
P. textilis textilinin 2a	MSSGGLLLLLGLLTLWEVLTPVSSKDRPELCELPPDTGPCRVRFPSFYYNPDEQKCLEFIYGGCEGNANNFITKEECESTC--AA
P. textilis textilinin 2b	MSSGGLLLLLGLLTLWEVLTPVSSKDRPELCELPPDTGPCRVRFPSFYYNPDEQKCLEFIYGGCEGNANNFITKEECESTC--AA
P. textilis textilinin 3	MSSGGLLLLLGLLTLWEVLTPVSSKDRPNFCKLPAETGRCNAKIPRFYYNPRQHQCIEFLYGGCGGNANNFKTIKECESTC--AA
P. textilis textilinin 7	MSSGGLLLLLGLLTLWEVLTPVSSKDRPKFCELLPDTGSCEDFTGAFHYSTRDRECIEFIYGGCGGNANNFKTLEECESTC--AA
O. microlepidotus microlepidin 5	MSSGGLLLLLGLLTLWEVLTPVSSKDRPKFYELPADIGPCEDFTGAFHYSPREHECIEFIYGGCEGNANNFNTLEECET------
O. microlepidotus microlepidin 3	MSSGGLLLLLGLLTLWEVLTPVSSKDRPKFCELPADIGPCEDFTGAFHYSPREHECIEFIYGGCEGNANNFNTLEECESAC--AA
N. scutatus tigerin 3	MSSGGLLLLLGLLTLWEILTPVSSKDRPHFCHLPHDTGPCKRNIQAFYYNPVYHTCLKFIYGGCQGNSNNFKTIDECKRTC--AA
N. scutatus tigerin 4	MSSGGLLLLLGLLTLWAELTPVSSKDHPEFCELPADSGPCRGILRAFYYHPVHRTCQMFIYGGCYGNANNFKTIDECKRTC--AA
N. scutatus tigerin 1	MSSRGLLLLLGLLTLWAELTPVSSKDHPEFCELPADSGPCRGILHAFYYHPVHRTCLEFIYGGCYGNANNFKTIDECKRTC--AA
B. candidus KTSPI A	MSSGGLLLLLGLLTLCAELTPVSSKDRPKFCNVPPEPGRCNANVRAFYYNPRLRKCIEFTYGGCGGNANNFKSRGECKRTC--AE
B. candidus KTSPI C	MSSGGLLLLLGLLTLWTELTPVSSKNRPPFCNLLPEPGRCNAIVRAFYYNSRLRKCLEFPYGGCGGNANNFKTIDECQRTC--AG
B. candidus β-bungarotoxin B2a	MSSGGLLLLLGLLTLWAELTPVSSRKRHPDCDKPPDTKICQTVVRAFYYKPSAKRCVQFRYGGCNGNGNHFKSDHLCRCECLEYP
B. candidus β-bungarotoxin B2b	MSSGGLLLLLGLLTLWAELTPVSSRKRHPDCDKAPDTRICQTVVRAFYYKPSEKRCVQFRYGGCKGNGNHFKSDHLCRCECLEYR
B. candidus β-bungarotoxin B1a	MSSGGLLLLLGLLTLWAELTPVSSRKRHPDCDKPPDKGNCGSVRRAFYYDTKLKTCKPFPYRGCKGNGNHFKTETLCRCECLVYP
B. candidus β-bungarotoxin B1b	MSSGGLLLLLGLLTLCAELTPVSSRQRHRDCDKPPDKGNCGSVRRAFYYDTRLKTCKAFPYRGCNGNGNHFKTETLCRCECLVYP
B. candidus PILP1a	MSSGSLLLLLGLLTFWAQLTPVSTKDRP-NCDKAPDTERCKPNVHAFYYNPSARDCLQFVYGGCDGNGKHFRSKALCLFHC--HR
B. candidus PILP1b	MSSGGLLLLLGLLTLWAELTPVSSRQRH-HCDKAPDTGRCKPNVHAFYYNPSARDCLQFVYGGCDGNGKHFRSKALCLFHC--HR
B. candidus B5	MSSGGLLLLLGLLTLCAELTPVSSKDRHPYCNLPPDPGPCHDNKFAFYHHPASNKCKEFVYGGCGGNDNRFKTRNKCQCTC--SG

Fig. S14. Australian elapid Kunitz-type toxins possess key residues associated with Kv1 potassium channel binding. Amino acid sequence alignment of hypothetical protein translations of Kunitz-type toxin genes**.** Residues shown in grey encode the signal peptide, and residues associated with plasmin inhibition or specificity are highlighted in red (7), whereas residues associated with Kv1 potassium-channel binding are highlighted in blue (8). Note that the β-bungarotoxin paralogs are known potassium-channel inhibiting neurotoxins (9, 10).

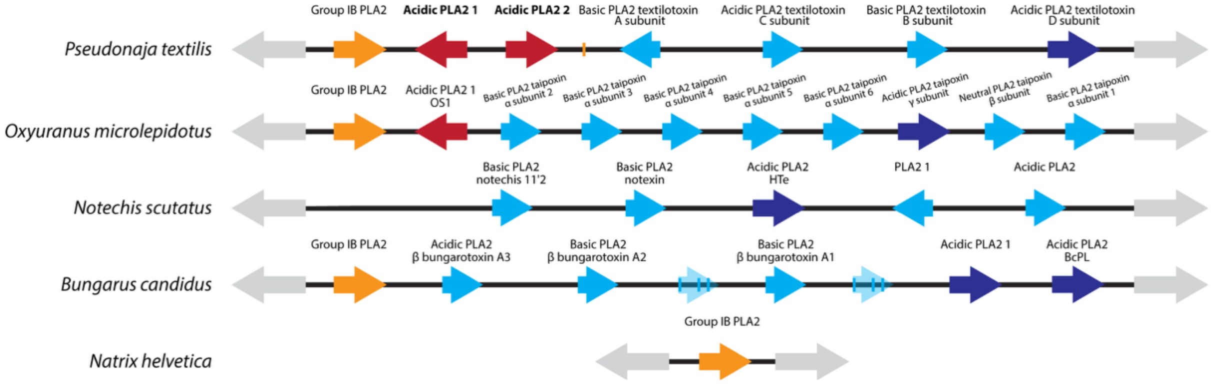

Fig. S15. Venom-expressed neurotoxic PLA2GIB proteins were neofunctionalized into procoagulant toxins. Protein identifications for the group I phospholipase genes shown in Fig. 5A, including procoagulant PLA2GIB proteins (red arrows), PLA2GIB proteins with neurotoxic or unknown activity (dark blue), neurotoxic PLA2GIA proteins (light blue), and the non-venom-expressed group IB PLA2 ortholog (orange). The *O. microlepidotus* Acidic PLA2 1 OS1 is a possible procoagulant protein based on its orthologous relationship with *P. textilis* Acidic PLA2 1; however, this protein has not been biochemically characterized.

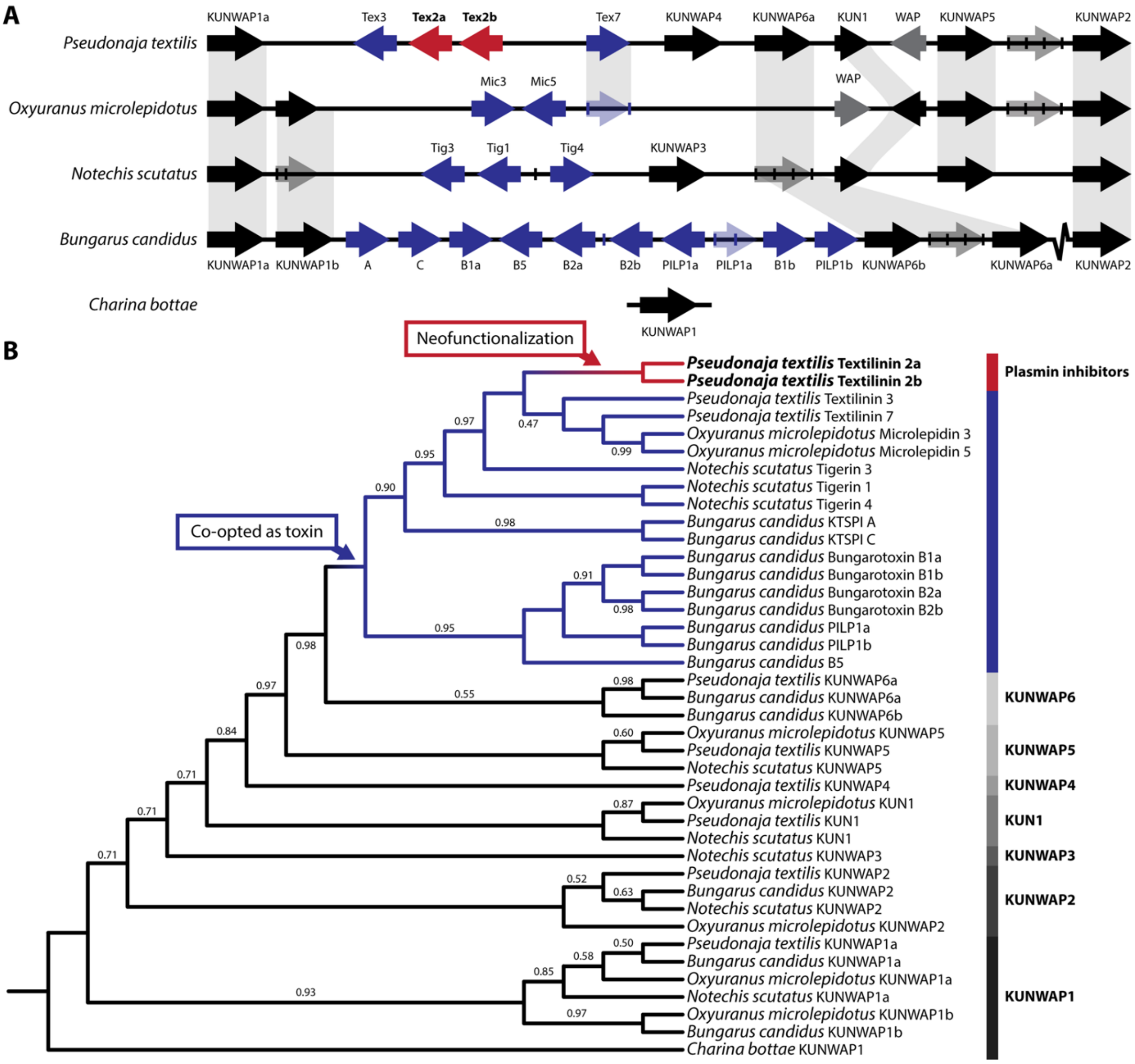

Fig. S16. Venom-expressed Kunitz-type toxins were neofunctionalized into plasmin-inhibiting proteins.

(A) A schematic showing the synteny of venom-expressed and non-venom-expressed Kunitz-type serine protease inhibitor genes across different elapid snakes. Pseudogenes are indicated by transparent arrows. The break-line symbol represents a genomic region with low confidence.

(B) A protein phylogeny of Kunitz-type serine protease inhibitor genes. Full phylogeny of Figure 6, with all Kunitz–Waprin protein (KUNWAP) sequences. The plasmin-inhibiting proteins (Textilinin 2a and Textilinin 2b) cluster within the clade of all venom-expressed genes. The phylogeny was rooted with the single-copy gene of *C. bottae* as the outgroup. Only Bayesian posterior probabilities less than 1.00 are shown.

Tables

Table S1. Comparison of gene expression levels between venom gland and liver tissues in *Pseudonaja textilis* using short-read RNA-sequencing.

Gene ID	Protein ID	Gene complex	Venom gland (TPM)	Liver (TPM)
NF2	Merlin	PLA2	69,59	34,15
PLA2G1B	Group IB PLA2	PLA2	3,99	0
PLA-1	Acidic PLA2 1	PLA2	140262,47	120,94
PLA-2	Acidic PLA2 2	PLA2	97315,99	99,91
PLA-3	Basic PLA2 textilotoxin A subunit	PLA2	23292,45	3
PLA-4	Acidic PLA2 textilotoxin C subunit	PLA2	42512,56	6
PLA-5	Basic PLA2 textilotoxin B subunit	PLA2	13785,71	2,87
PLA-6	Acidic PLA2 textilotoxin D subunit	PLA2	27505,92	0
THOC5	THO complex subunit 5	PLA2	394,56	335,28
KUNWAP1a	Kunitz–Waprin protein 1a	KUN	12,16	314,3
Textilinin-3	Textilinin 3	KUN	12137,17	0
Textilinin-2a	Textilinin 2a	KUN	274025,93	167,99
Textilinin-2b	Textilinin 2b	KUN	274025,93	167,99
Textilinin-7	Textilinin 7	KUN	49246,97	48
KUNWAP4	Kunitz–Waprin protein 4	KUN	23,94	0
KUNWAP6a	Kunitz–Waprin protein 6a	KUN	0	0
KUN1	Kunitz protein 1	KUN	0	0
WAP1	Waprin 1 (Textwaprin)	KUN	0	0
KUNWAP5	Kunitz–Waprin protein 5	KUN	0	0
KUNWAP2	Kunitz–Waprin protein 2	KUN	0	0

Abbreviation: TPM, Transcripts per Million. Similar TPM values were obtained using Kallisto (data not shown) (11).

Table S2. Comparison of gene expression levels between venom gland and liver tissues in the Inland taipan (*Oxyuranus microlepidotus*) using short-read RNA-sequencing.

Gene ID	Protein ID	Gene complex	Venom gland (TPM)	Liver (TPM)
NF2	Merlin	PLA2	6,94	152,88
PLA2G1B	Group IB PLA2	PLA2	0,47	0
PLA-1	Acidic PLA2 1 OS1	PLA2	22561,82	0
PLA-2	Basic PLA2 taipoxin α subunit 2	PLA2	176034,55	0,61
PLA-3	Basic PLA2 taipoxin α subunit 3	PLA2	176034,55	0,61
PLA-4	Basic PLA2 taipoxin α subunit 4	PLA2	166106,5	0,3
PLA-5	Basic PLA2 taipoxin α subunit 5	PLA2	166106,5	0,3
PLA-6	Basic PLA2 taipoxin α subunit 6	PLA2	96369,47	0
PLA-7	Acidic PLA2 taipoxin γ subunit	PLA2	74955,84	0
PLA-8	Neutral PLA2 taipoxin β subunit	PLA2	53611,41	0
PLA-9	Basic PLA2 taipoxin α subunit 1	PLA2	42857,19	0
THOC5	THO complex subunit 5	PLA2	31,85	1273,86
KUNWAP1a	Kunitz–Waprin protein 1a	KUN	0	0
KUNWAP1b	Kunitz–Waprin protein 1b	KUN	0	0
Microlepidin-3	Microlepidin 3	KUN	880,78	0
Microlepidin-5	Microlepidin 5	KUN	218,67	0
WAP1	Waprin 1 (Omwaprin)	KUN	20991,74	0
KUN1	Kunitz protein 1	KUN	0,81	0
KUNWAP5	Kunitz–Waprin protein 5	KUN	1,98	0
KUNWAP2	Kunitz–Waprin protein 2	KUN	0	115,42

Abbreviation: TPM, Transcripts per Million. Similar TPM values were obtained using Kallisto (data not shown) (11).

SI References

1. A. M. Bolger, M. Lohse, B. Usadel, Trimmomatic: a flexible trimmer for Illumina sequence data. *Bioinformatics* **30**, 2114-2120 (2014).
2. H. Li, Aligning sequence reads, clone sequences and assembly contigs with BWA-MEM. *arXiv preprint arXiv:1303.3997* (2013).
3. P. Danecek *et al.*, Twelve years of SAMtools and BCFtools. *GigaScience* **10**, giab008 (2021).
4. J. T. Robinson *et al.*, Integrative genomics viewer. *Nature Biotechnology* **29**, 24-26 (2011).
5. H. Thorvaldsdóttir, J. T. Robinson, J. P. Mesirov, Integrative Genomics Viewer (IGV): high-performance genomics data visualization and exploration. *Briefings in Bioinformatics* **14**, 178-192 (2013).
6. M. A. Reza, T. N. Minh Le, S. Swarup, R. Manjunatha Kini, Molecular evolution caught in action: gene duplication and evolution of molecular isoforms of prothrombin activators in Pseudonaja textilis (brown snake). *Journal of Thrombosis and Haemostasis* **4**, 1346-1353 (2006).
7. E.-K. I. Millers *et al.*, The structure of Human Microplasmin in Complex with Textilinin-1, an Aprotinin-like Inhibitor from the Australian Brown Snake. *PLOS ONE* **8**, e54104 (2013).
8. S. Gasparini *et al.*, Delineation of the functional site of alpha-dendrotoxin: The functional topographies of dendrotoxins are different but share a conserved core with those of other Kv1 potassium channel-blocking toxins. *Journal of Biological Chemistry* **273**, 25393-25403 (1998).
9. C. G. Benishin, Potassium channel blockade by the B subunit of beta-bungarotoxin. *Molecular Pharmacology* **38**, 164-169 (1990).
10. P.-F. Wu, S.-N. Wu, C.-C. Chang, L.-S. Chang, Cloning and functional expression of B chains of β-bungarotoxins from Bungarus multicinctus (Taiwan banded krait). *Biochemical Journal* **334**, 87-92 (1998).
11. N. L. Bray, H. Pimentel, P. Melsted, L. Pachter, Near-optimal probabilistic RNA-seq quantification. *Nature Biotechnology* **34**, 525-527 (2016).
